# Supplementary figures and images for: Disentangling the determinants of transposable elements dynamics in vertebrate genomes using empirical evidences and simulations
Source: PLoS Genet. 2020 Oct 5;16(10):e1009082. doi: 10.1371/journal.pgen.1009082 (PMC7561263; doi:10.1371/journal.pgen.1009082)

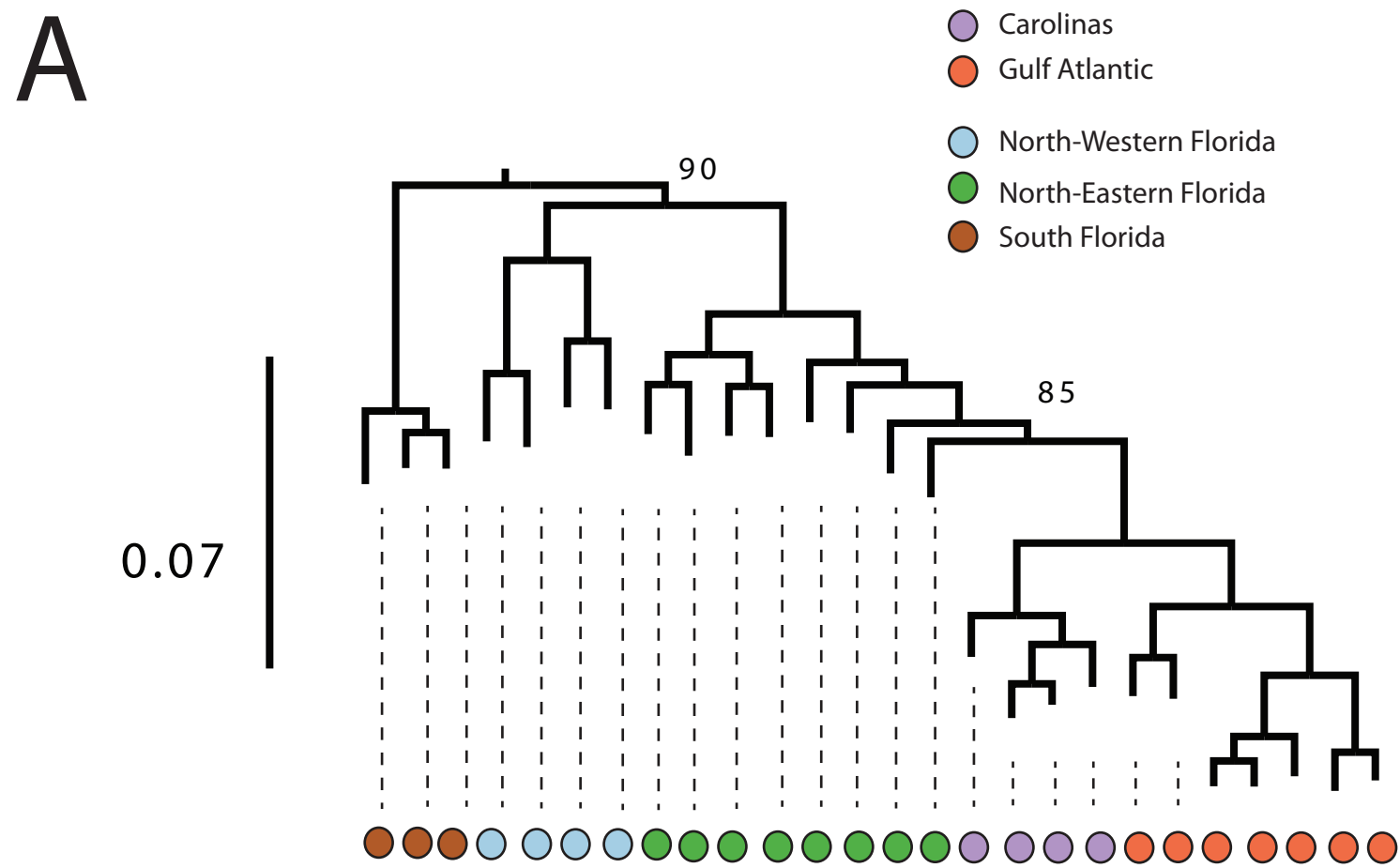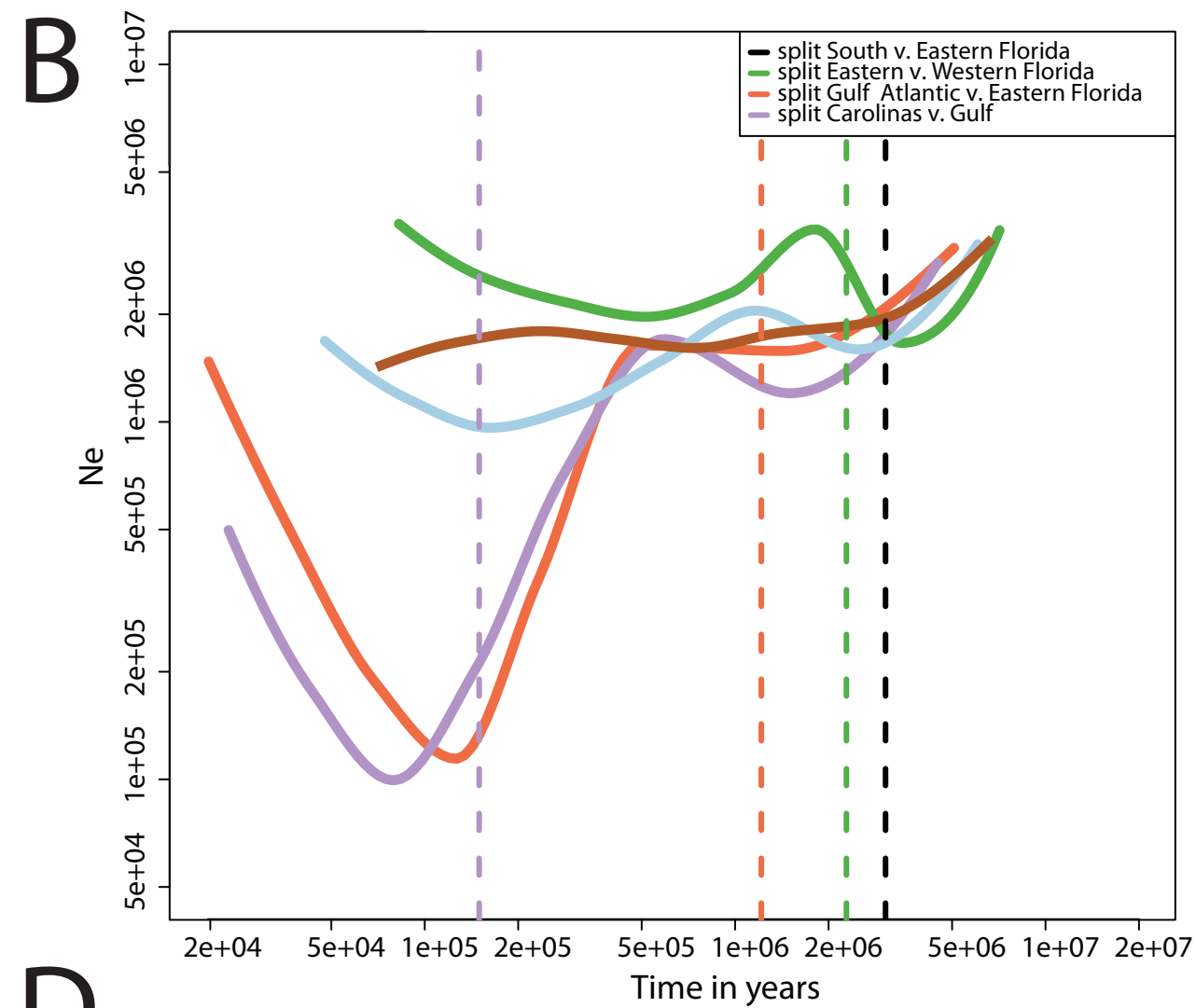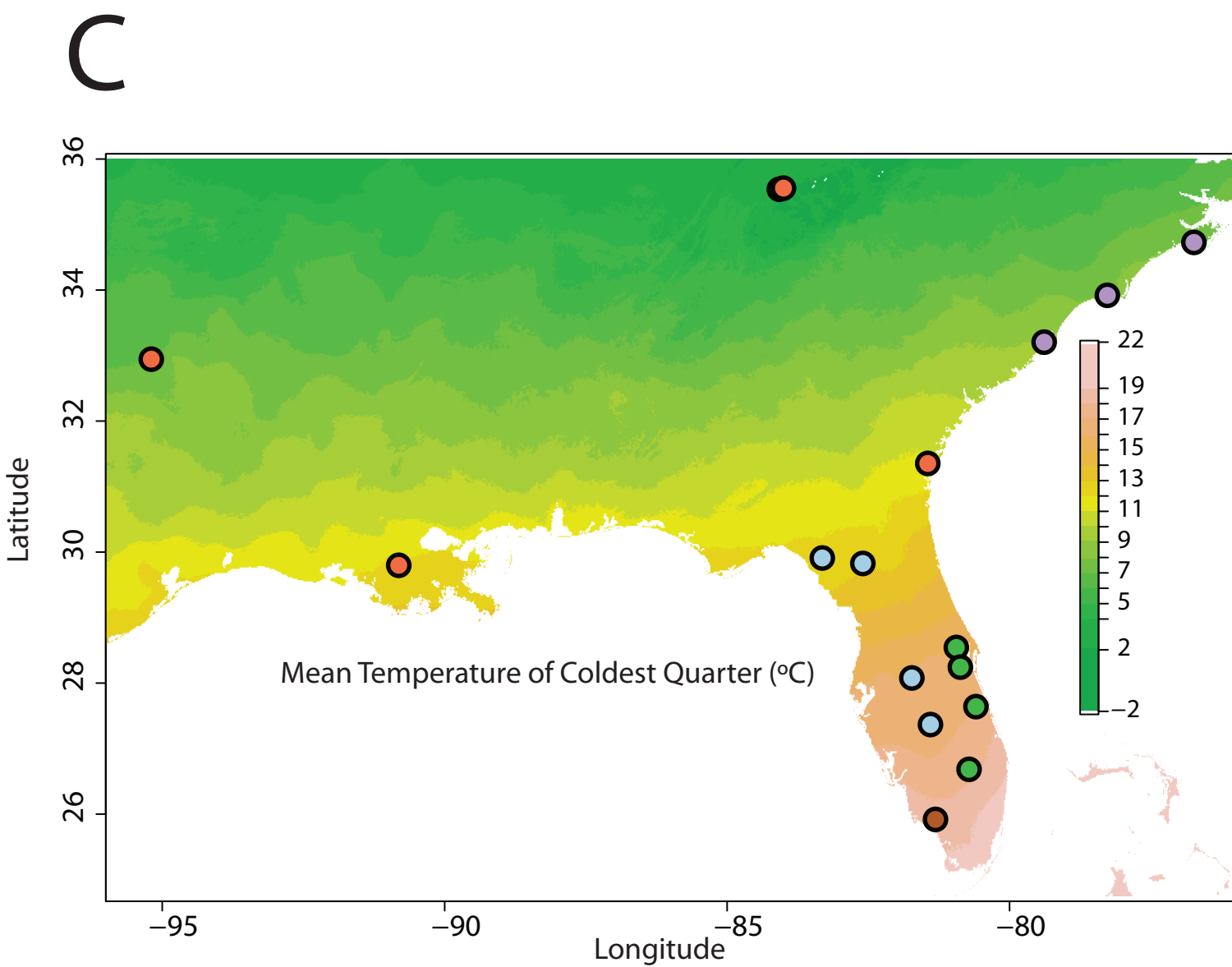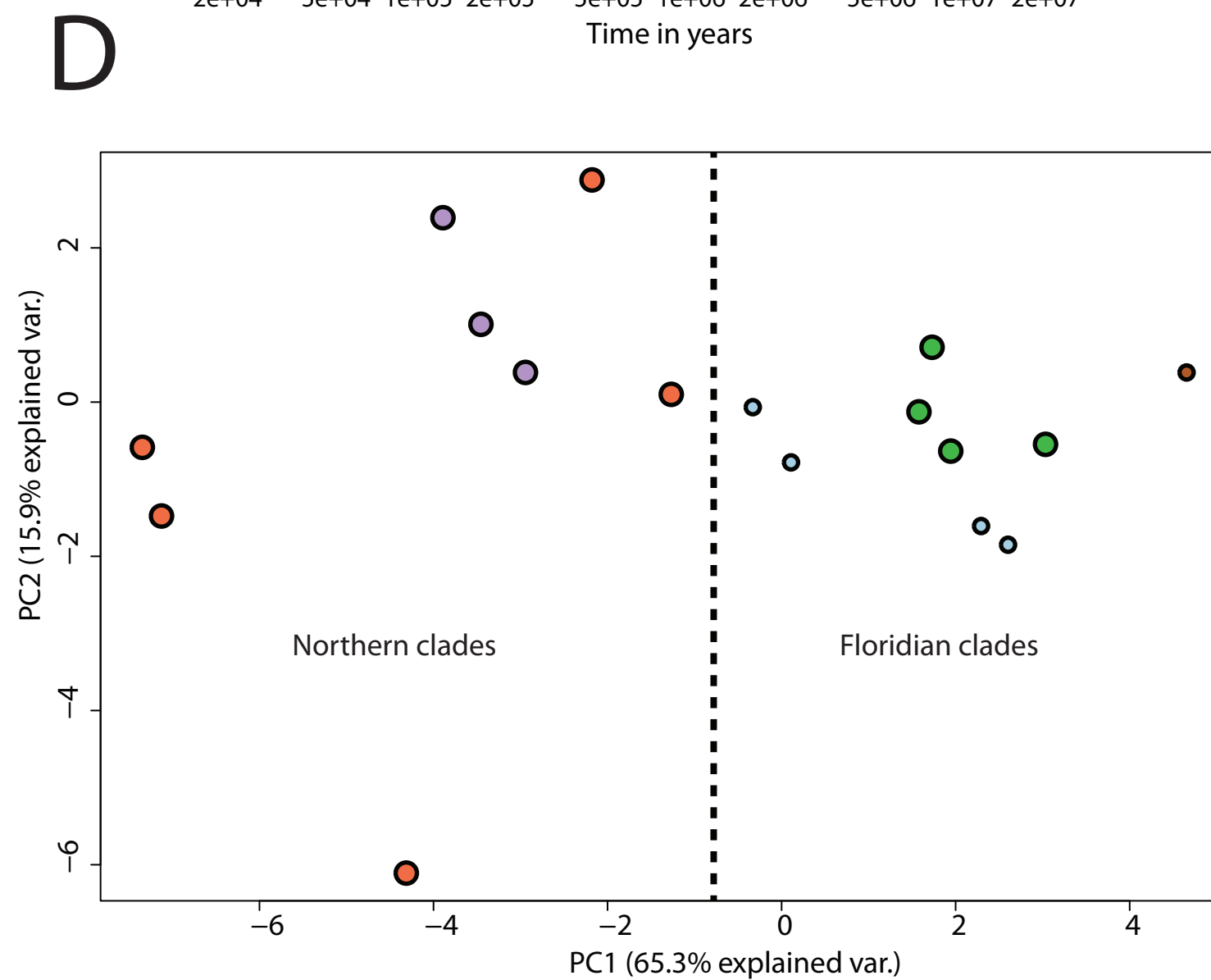

Supplement: S1 Fig — A: RAxML phylogeny on one million random SNPs. B: Demographic evolution of the five genetic clusters of green anoles reconstructed by SMC++ [82]. C: Sampling locations used in this study. Units for temperature are in tenth of Celsius degrees. D: PCA over environmental variables (BIOCLIM data) for the locations used in this study. Larger dots highlight the northern clades (GA and CA) and their sister Floridian clade (NEF). (PDF) [file pgen.1009082.s001.pdf]

NWF cluster

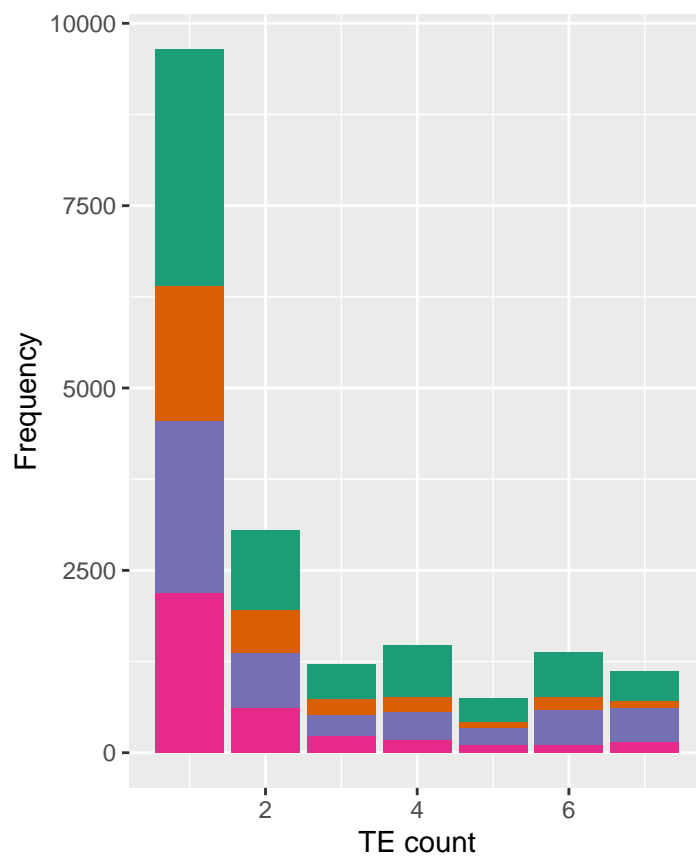

CA cluster

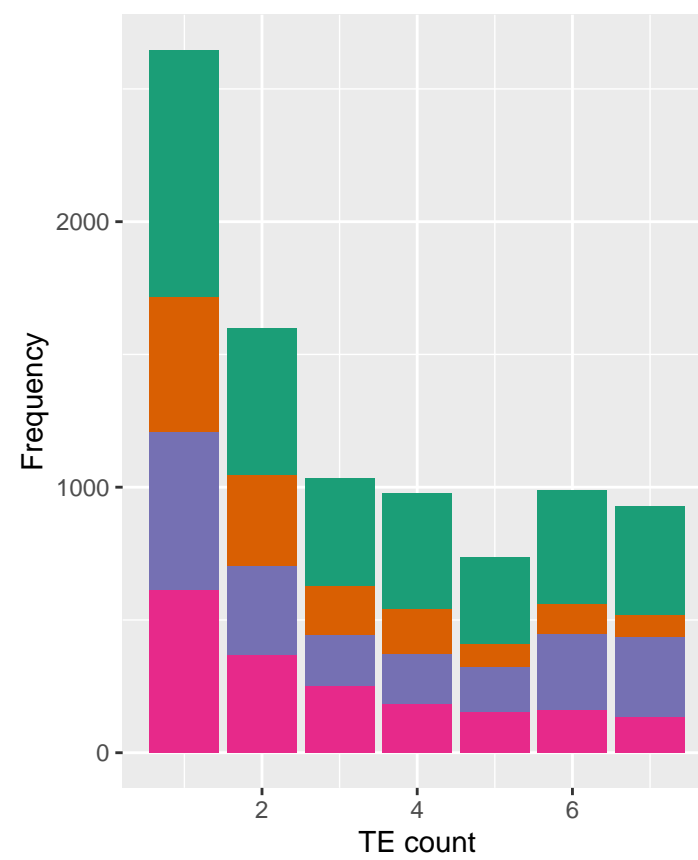

SF cluster

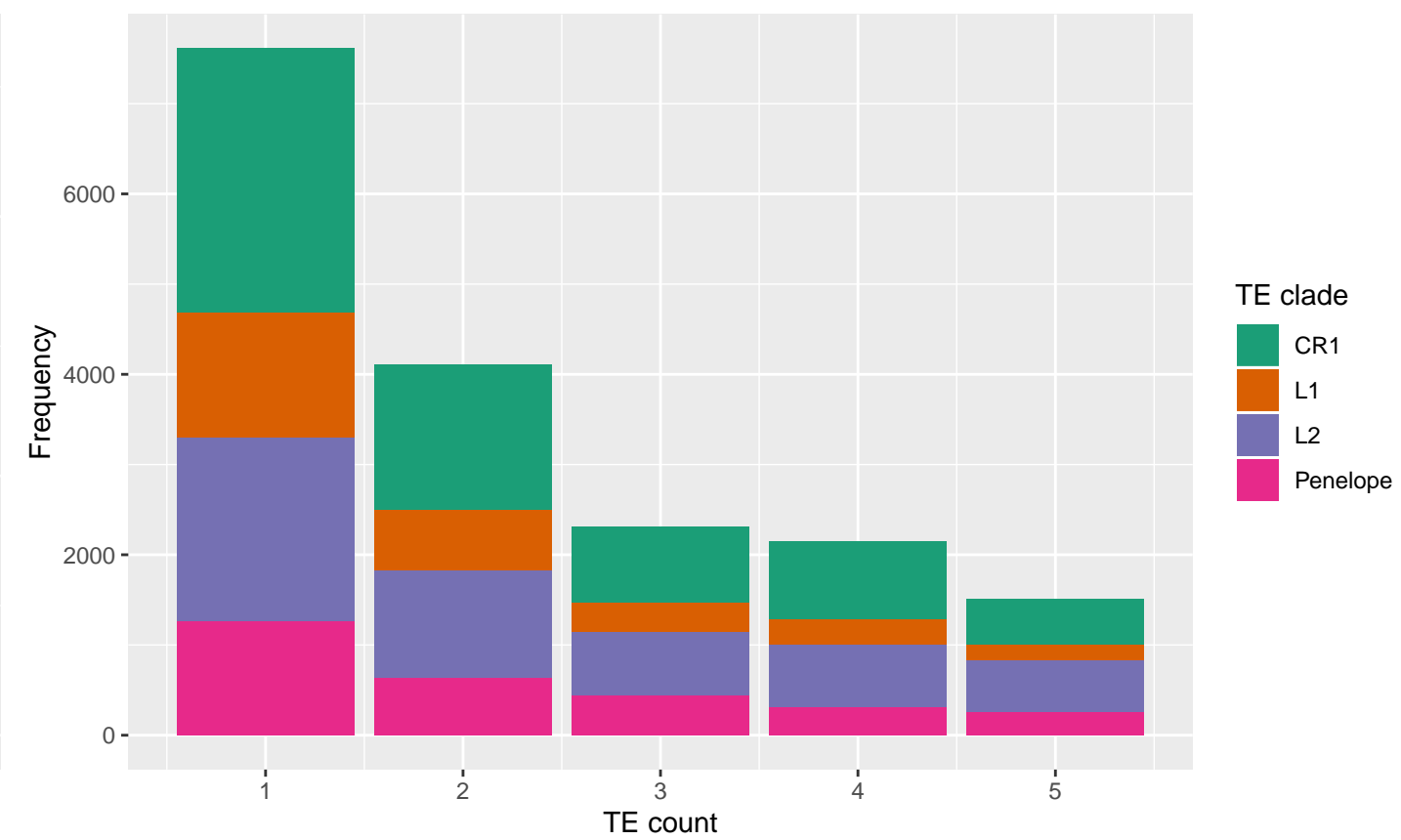

NEF cluster

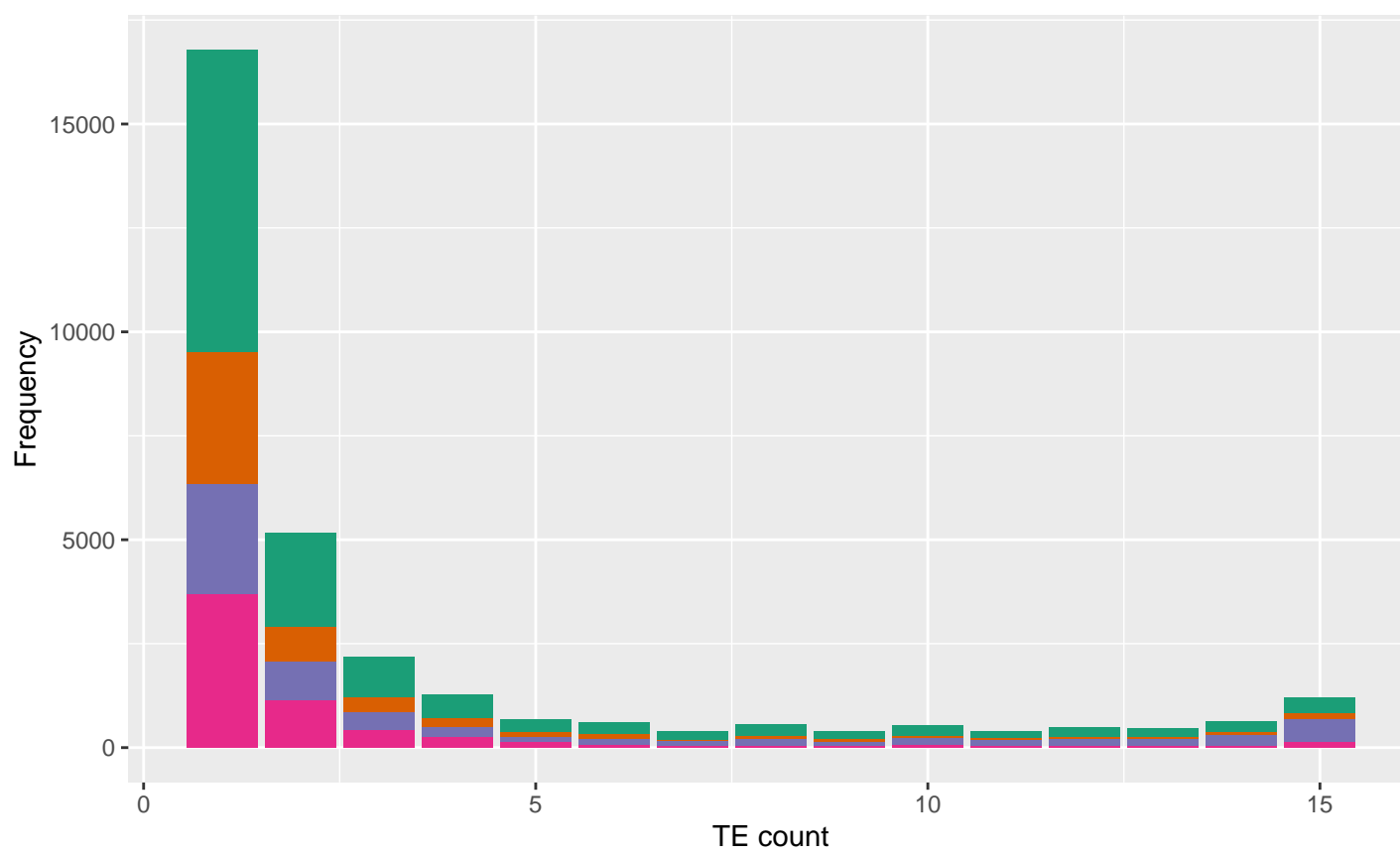

GA cluster

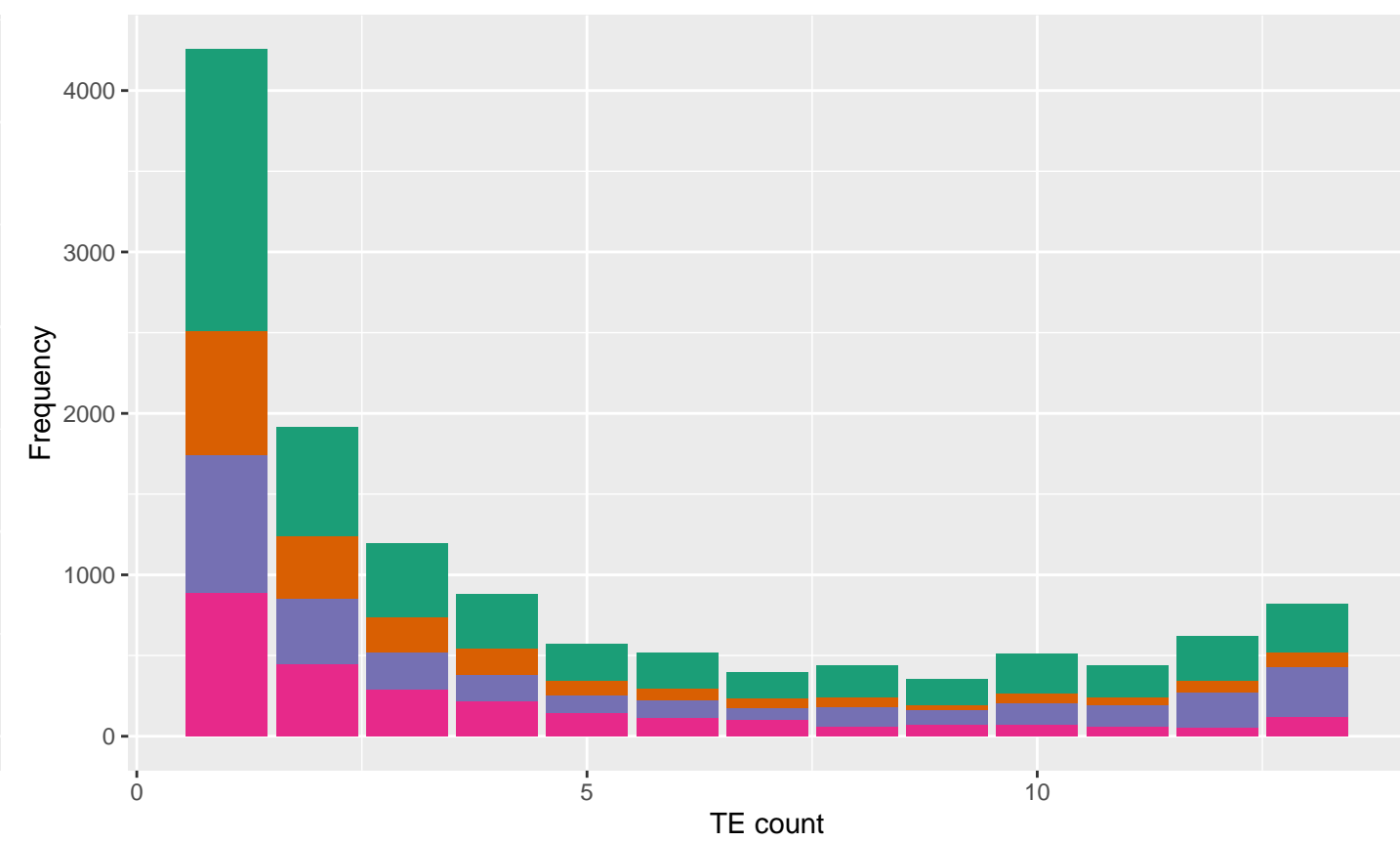

Supplement: S2 Fig — (PDF) [file pgen.1009082.s002.pdf]

NWF cluster

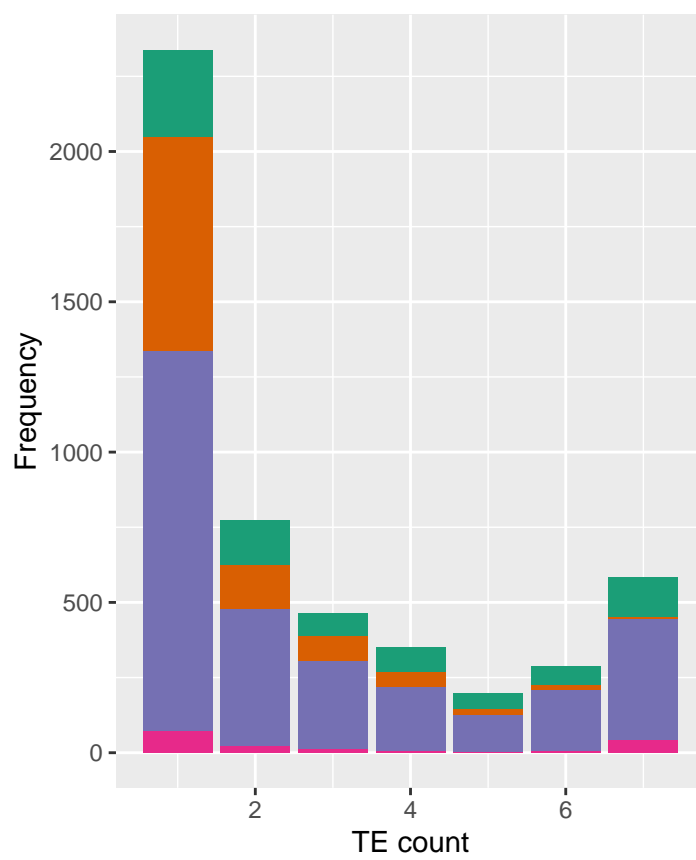

CA cluster

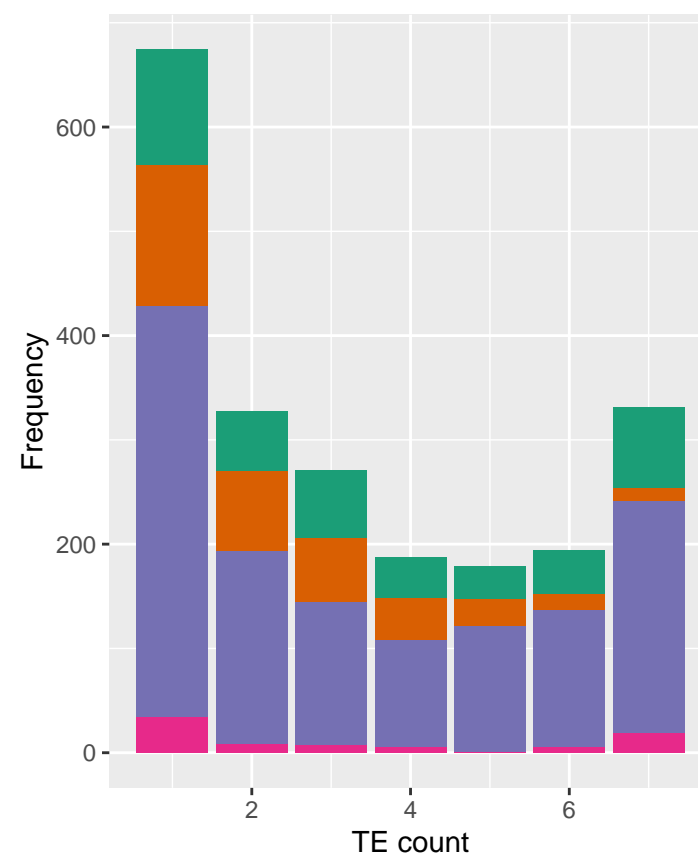

SF cluster

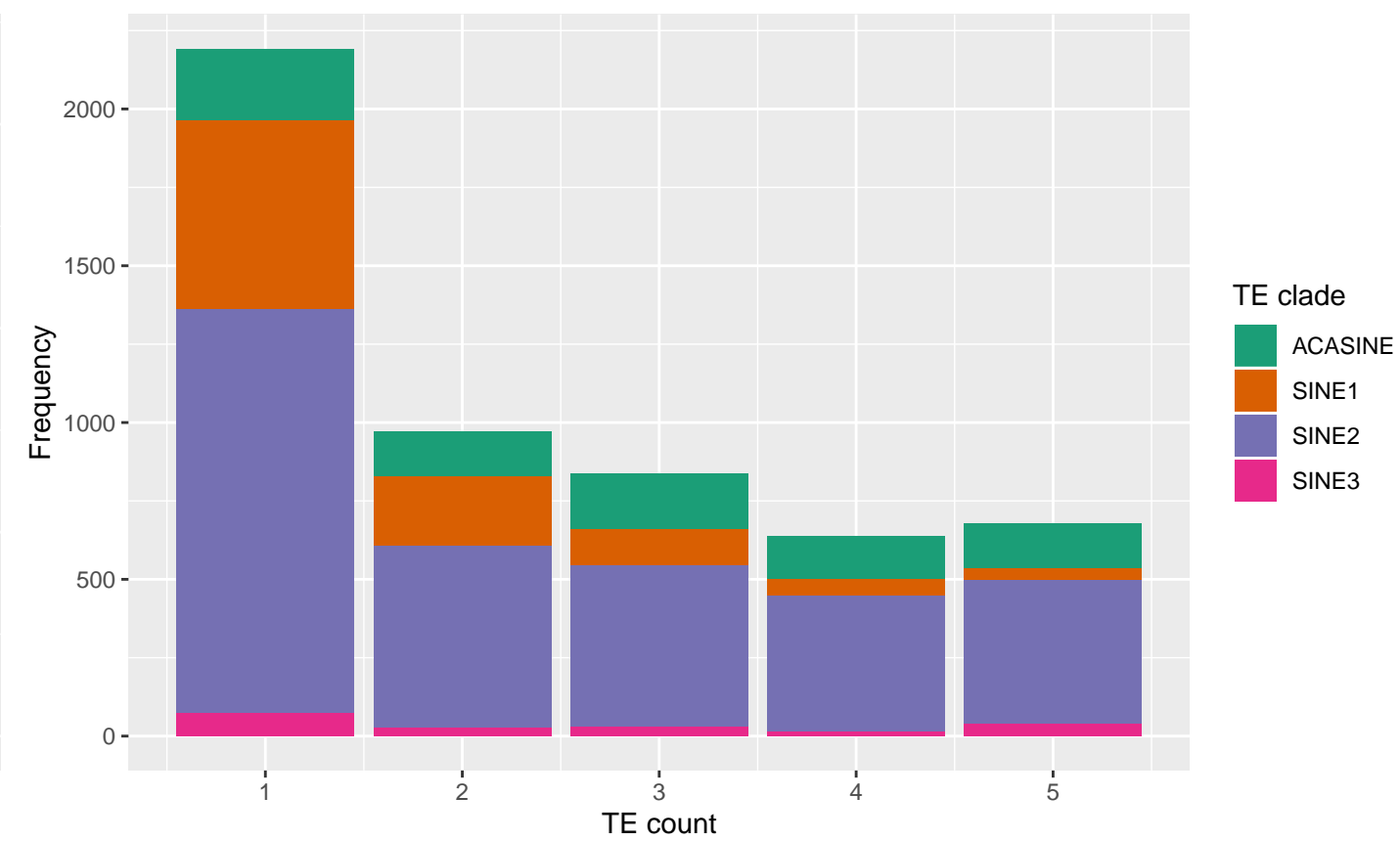

TE clade

- ACASINE
- SINE1
- SINE2
- SINE3

NEF cluster

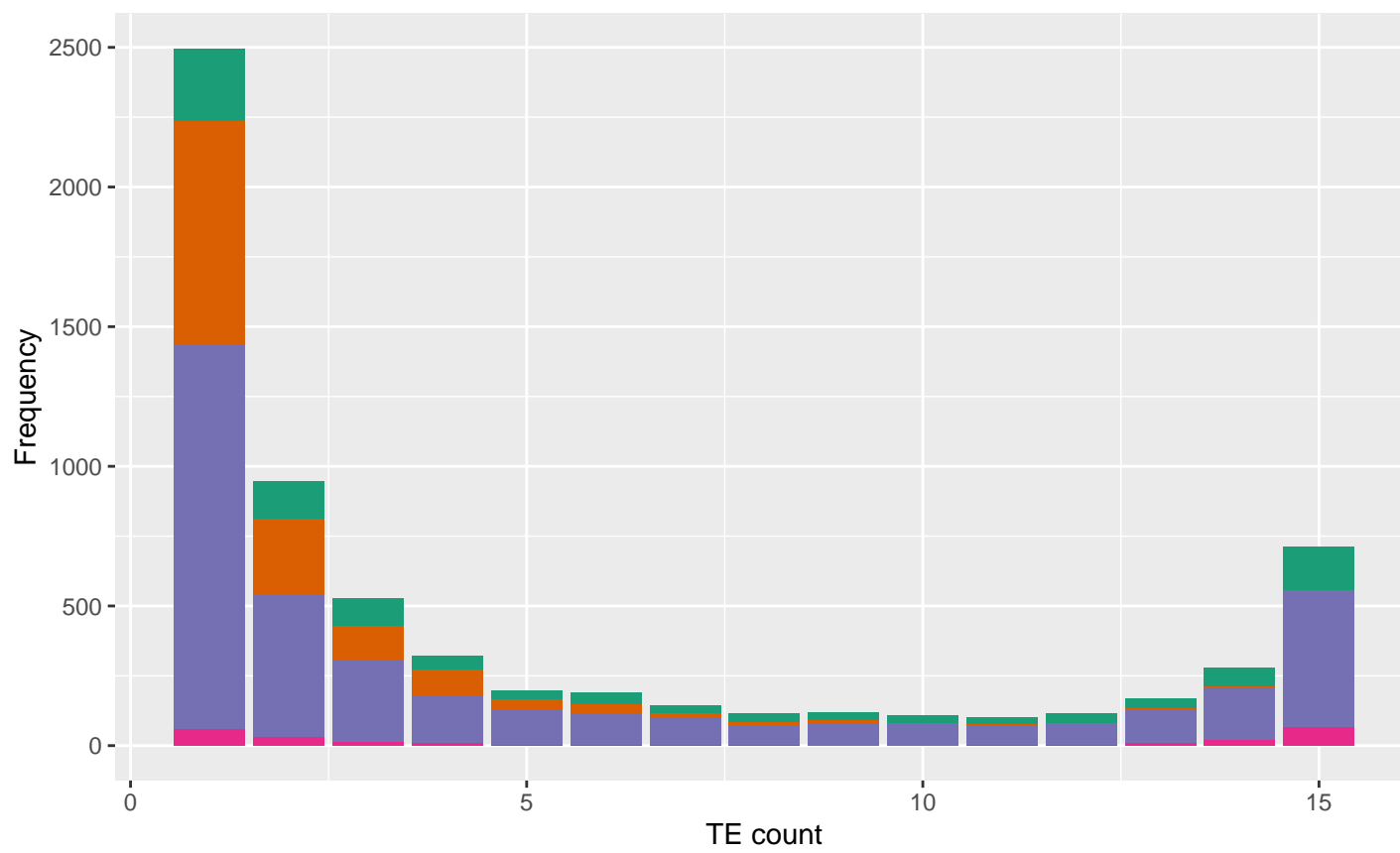

GA cluster

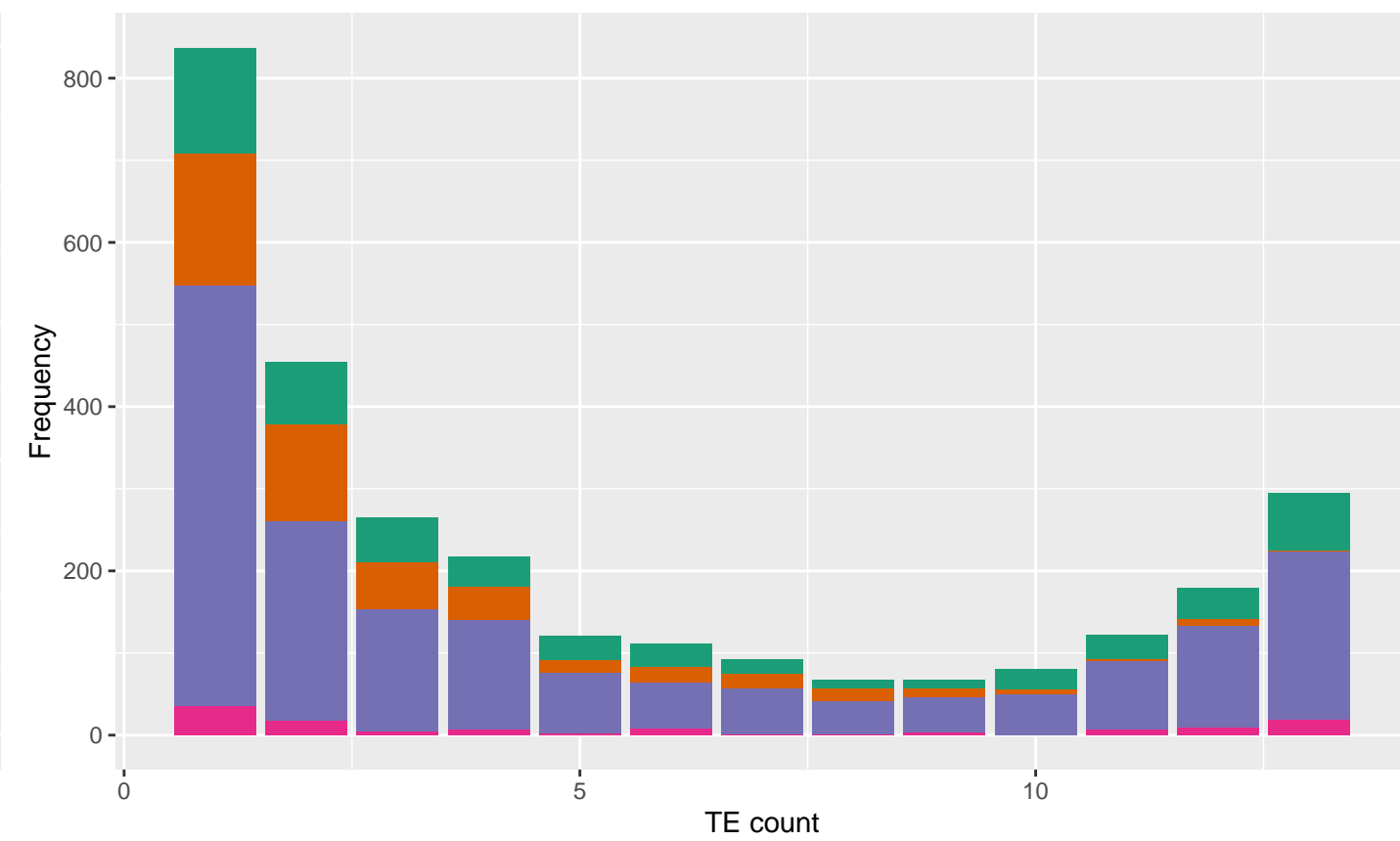

Supplement: S3 Fig — (PDF) [file pgen.1009082.s003.pdf]

NWF cluster

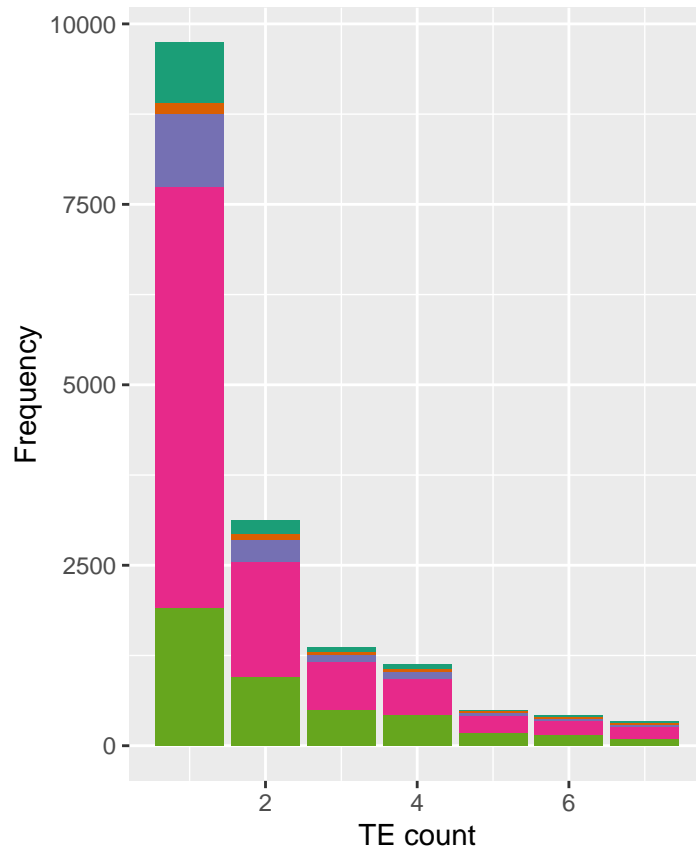

CA cluster

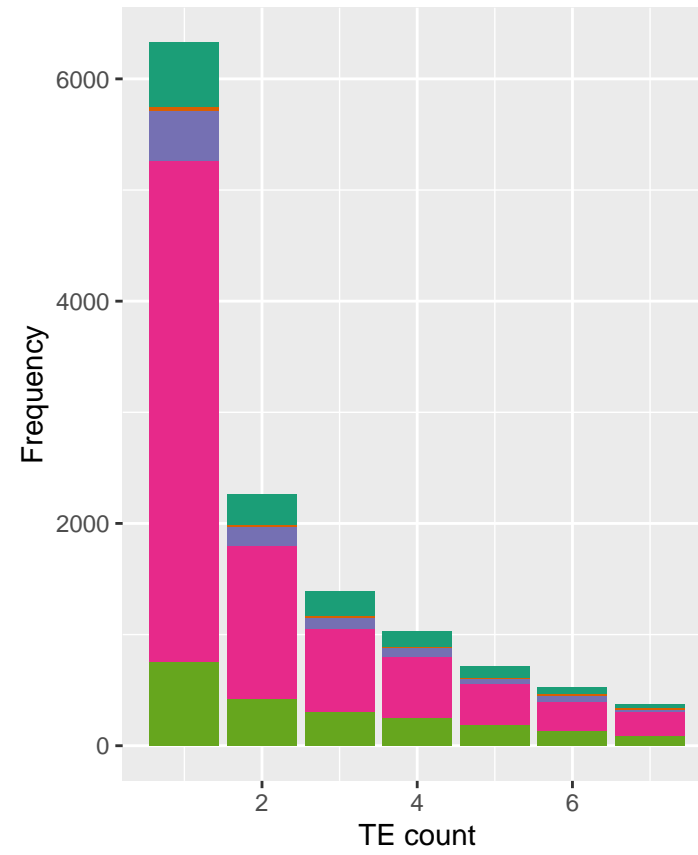

SF cluster

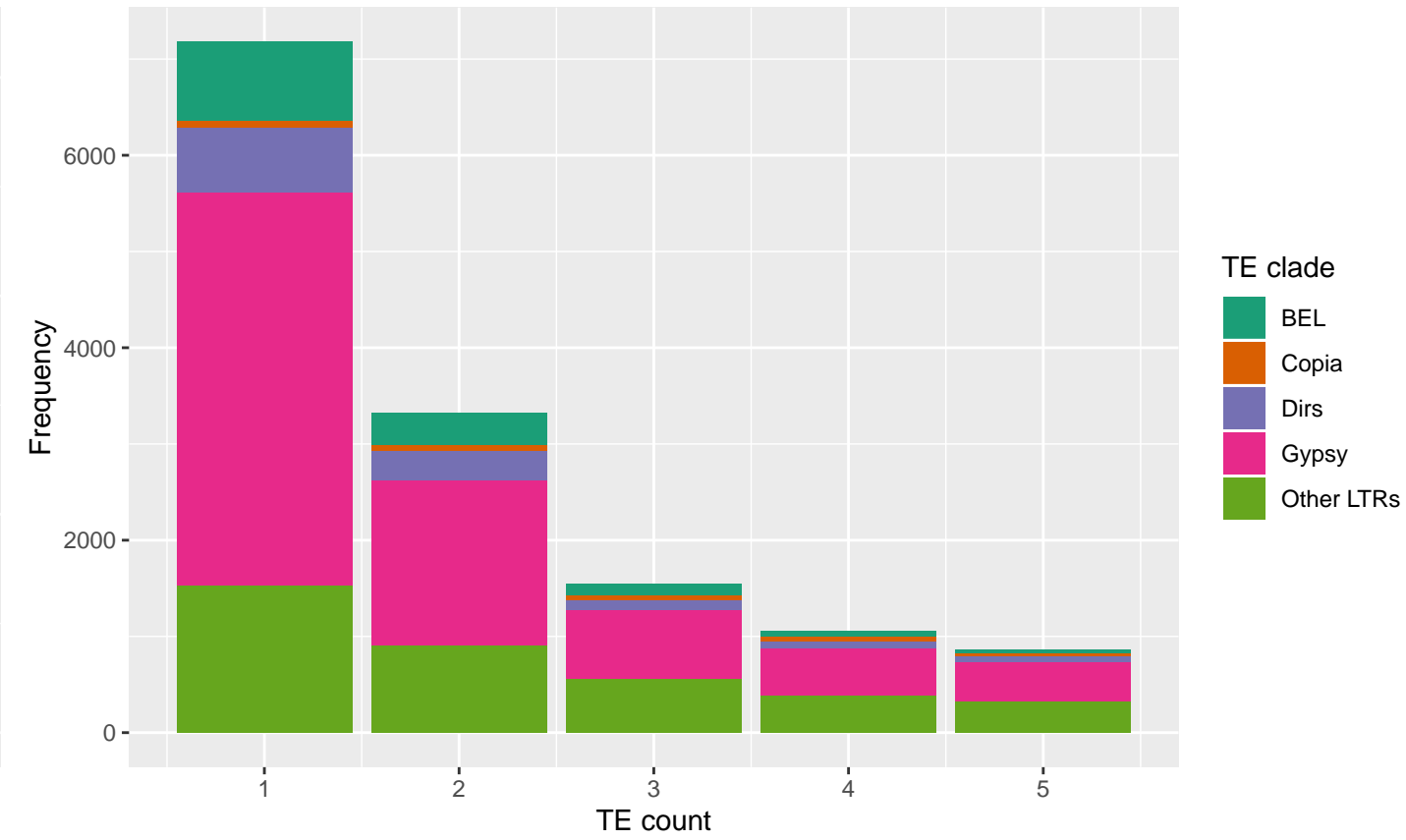

TE clade

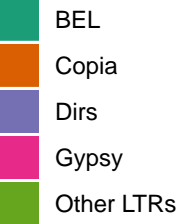

NEF cluster

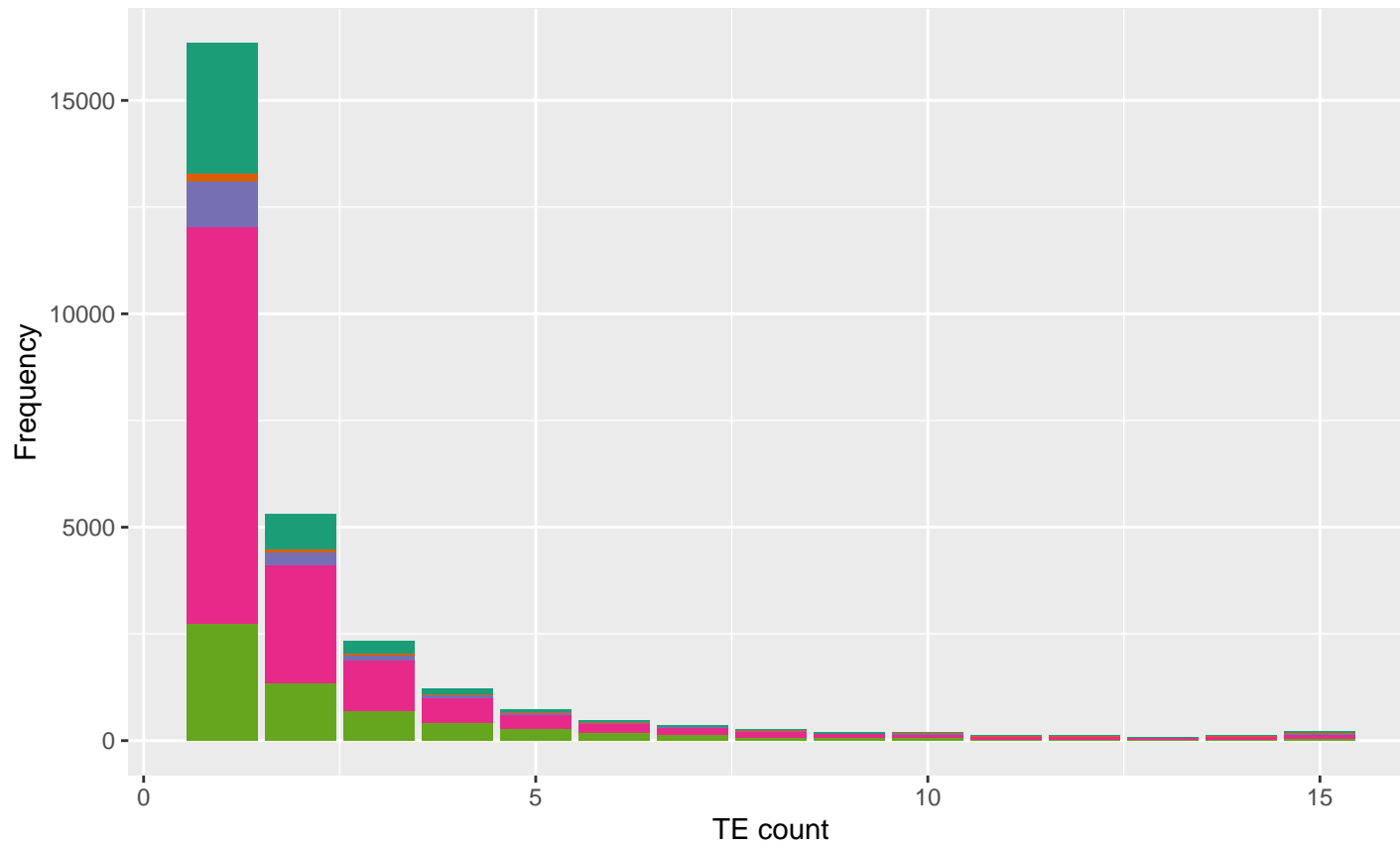

GA cluster

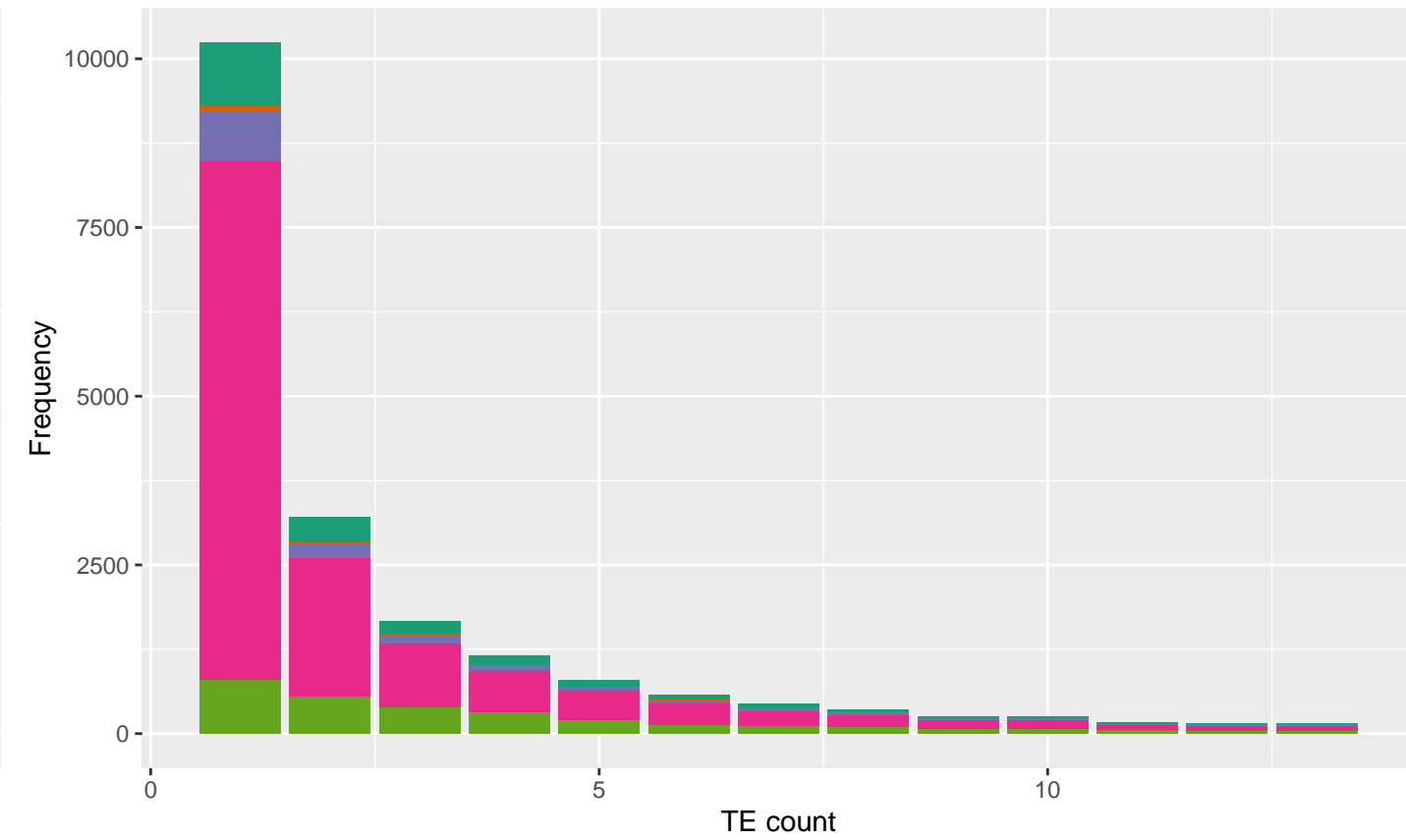

Supplement: S4 Fig — (PDF) [file pgen.1009082.s004.pdf]

NWF cluster

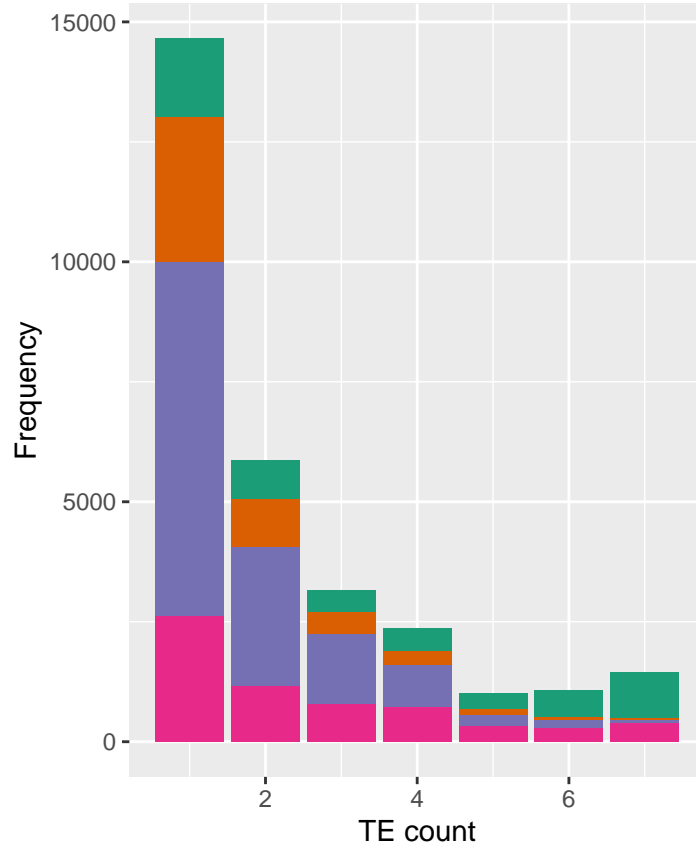

CA cluster

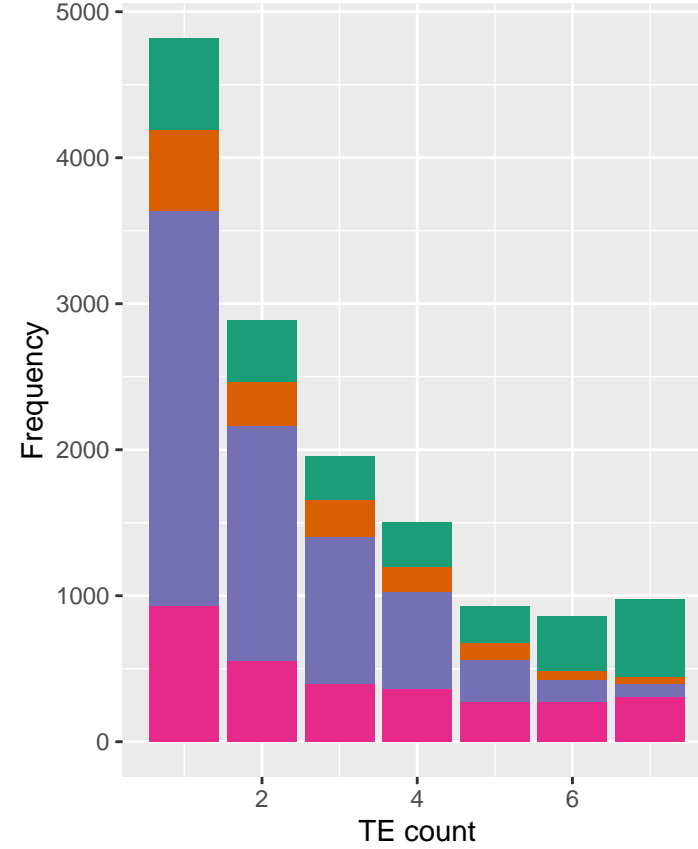

SF cluster

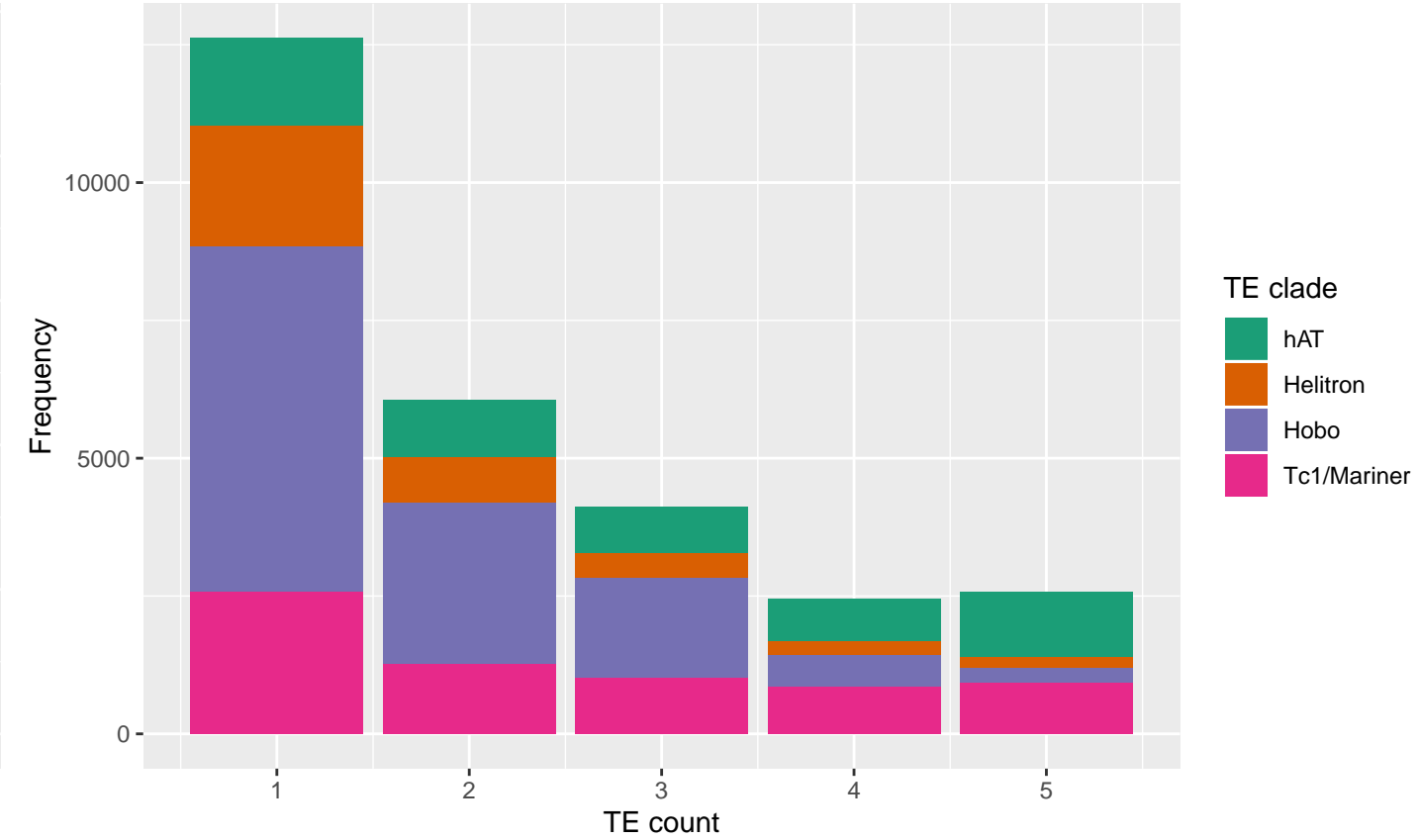

TE clade

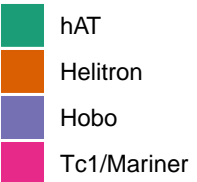

NEF cluster

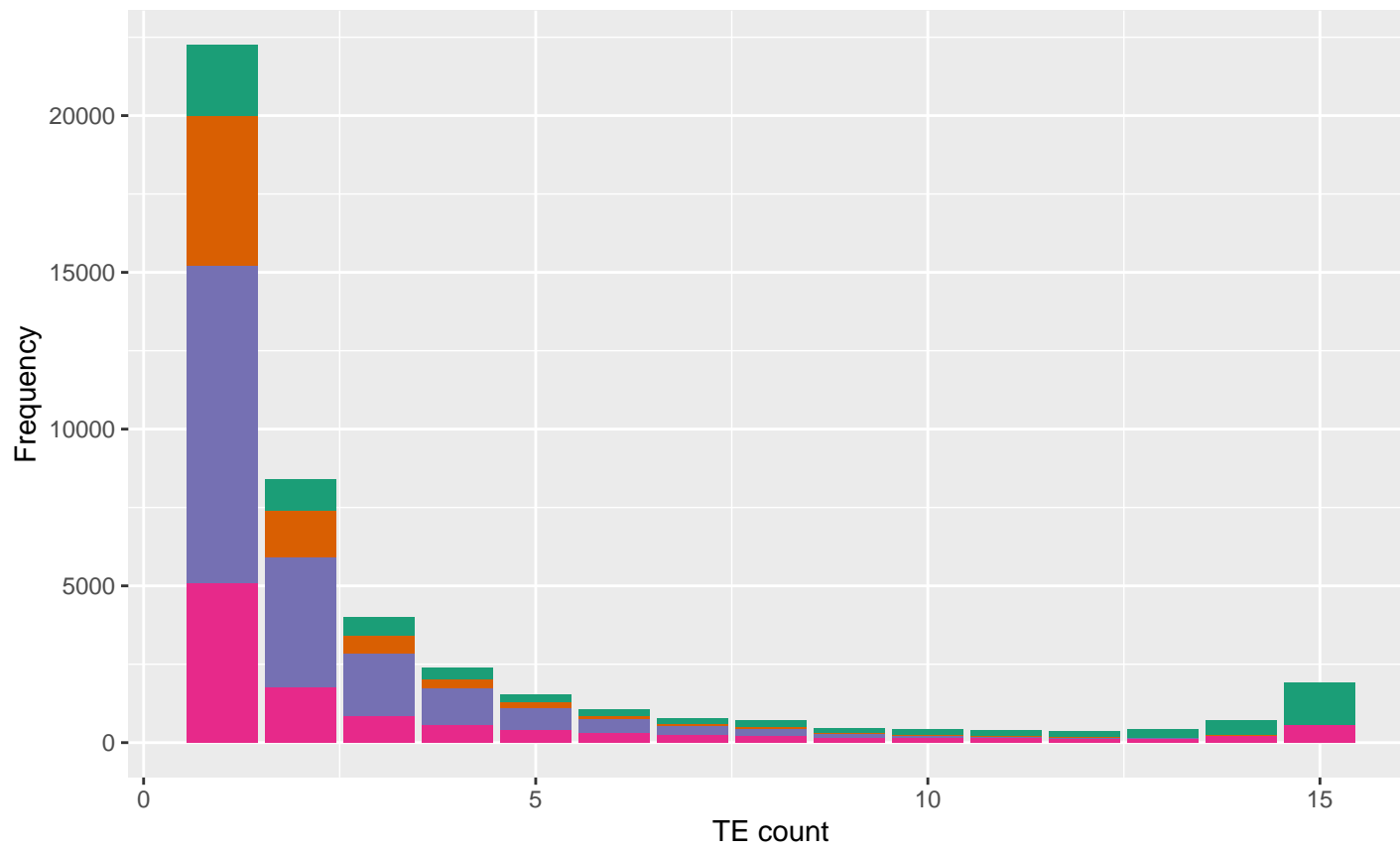

GA cluster

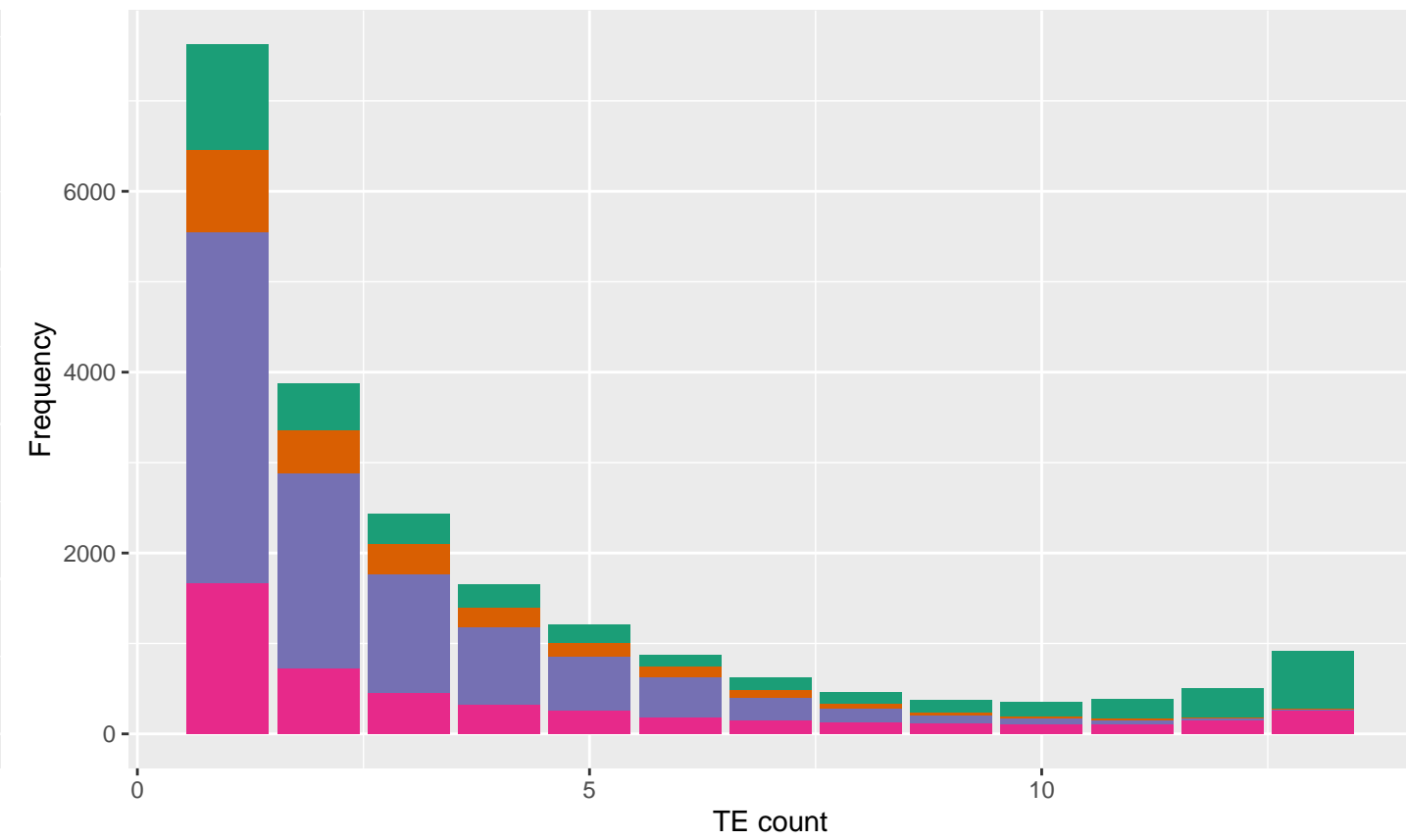

Supplement: S5 Fig — (PDF) [file pgen.1009082.s005.pdf]

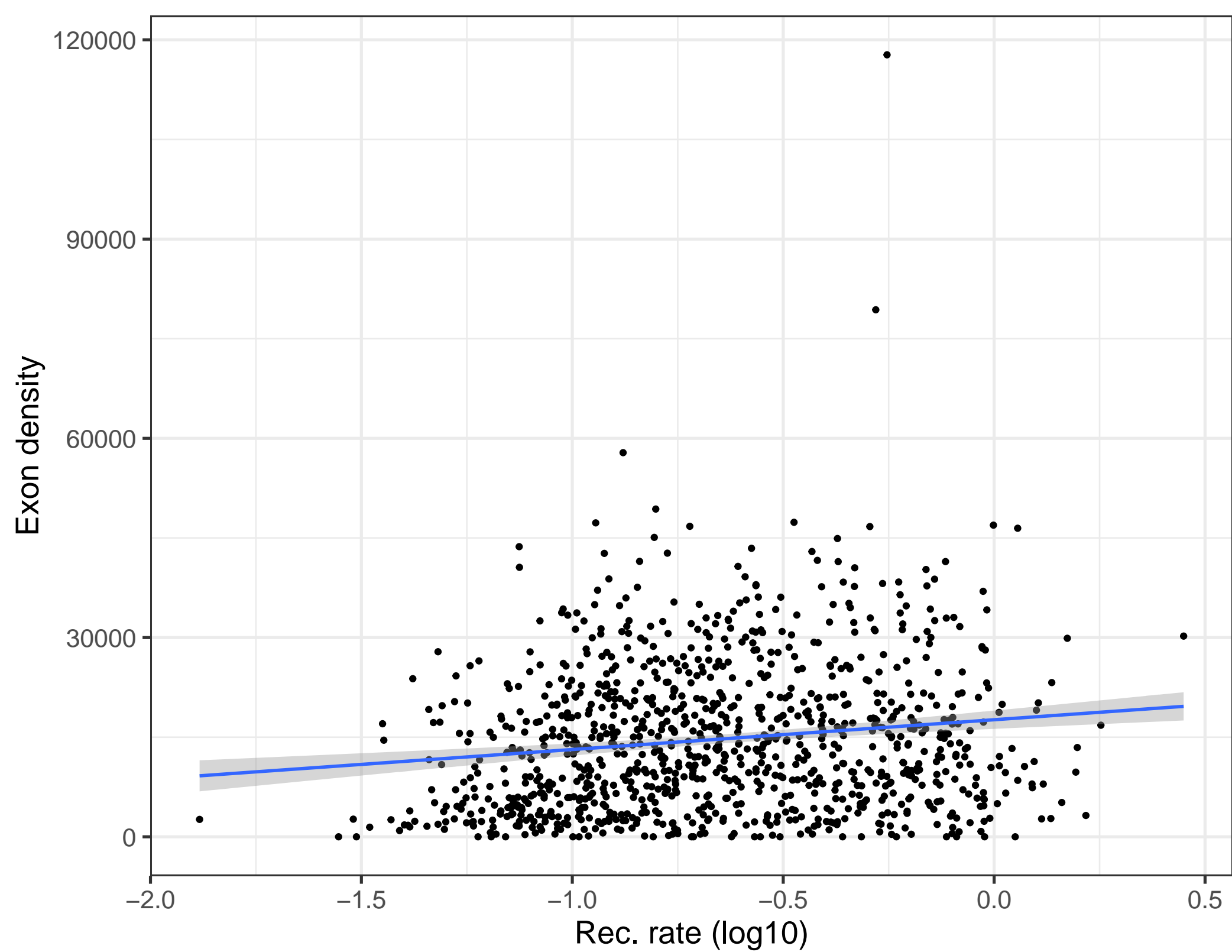

Supplement: S6 Fig — (PDF) [file pgen.1009082.s006.pdf]

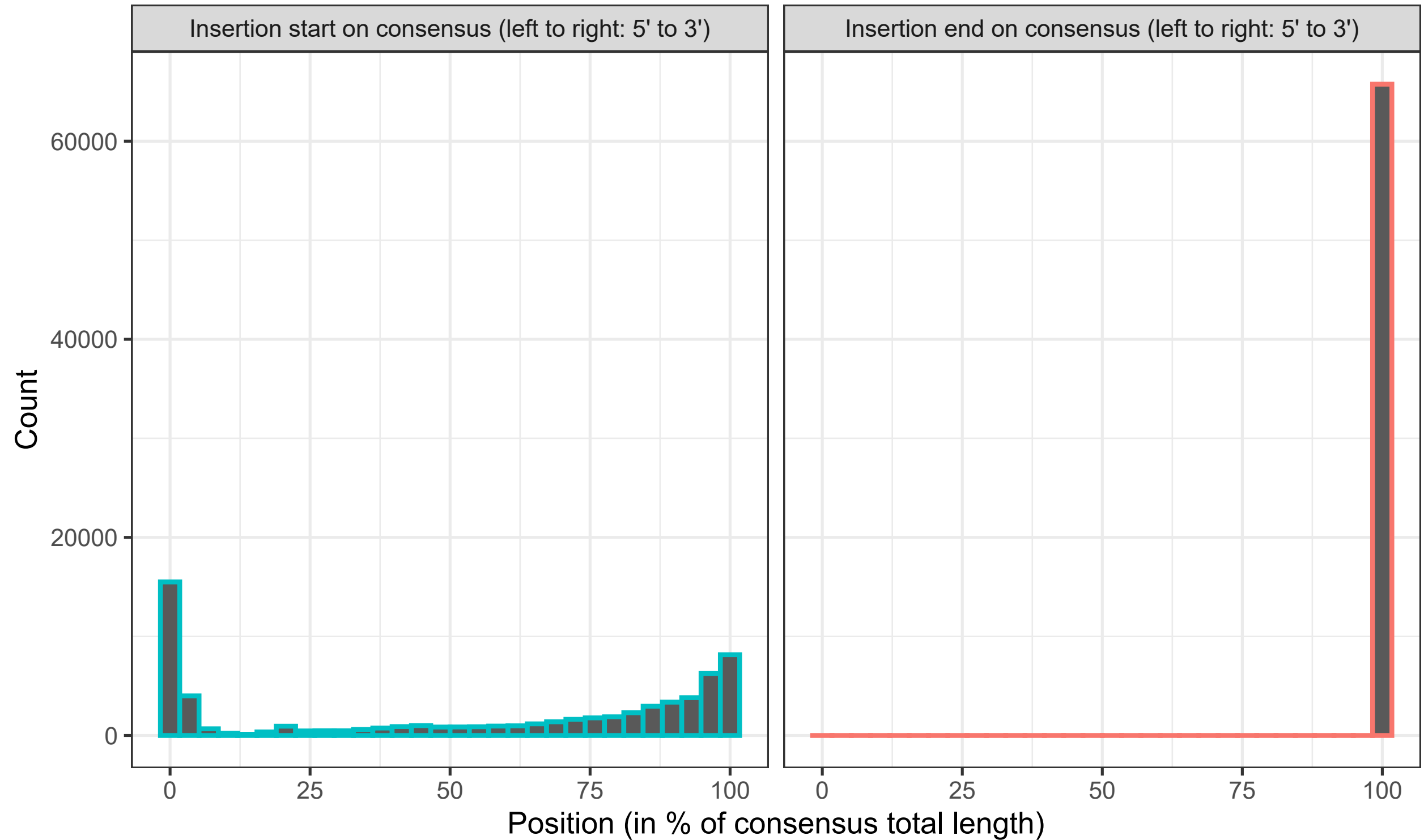

Supplement: S7 Fig — Left: position of the start of an element relative to its consensus, reflecting 5’ truncation. Right: position of the end of an element relative to its consensus. (PDF) [file pgen.1009082.s007.pdf]
