# Supplementary material for: Disentangling the determinants of transposable elements dynamics in vertebrate genomes using empirical evidences and simulations
Source: PLoS Genet. 2020 Oct 5;16(10):e1009082. doi: 10.1371/journal.pgen.1009082 (PMC7561263; doi:10.1371/journal.pgen.1009082)
Supplement: S8 Fig — Legend is the same as Fig 9. Parameters: 2Nes = -40 for 10% of non-coding sites and 2Nes = -400 for 70% of coding sites. (PDF) [file pgen.1009082.s008.pdf]

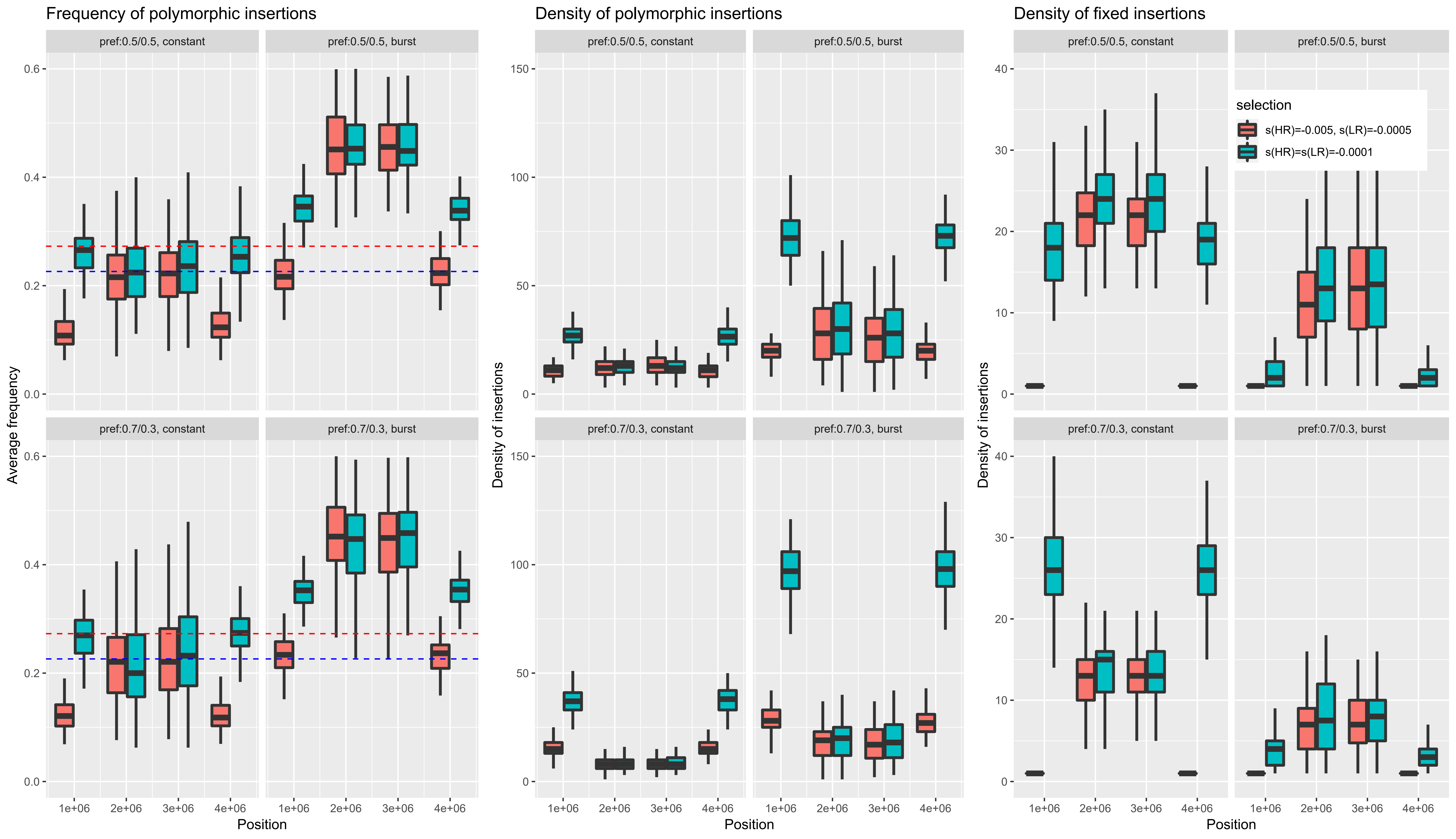
Frequency of polymorphic insertions
Density of polymorphic insertions
Density of fixed insertions
pref:0.5/0.5, constant
pref:0.5/0.5, burst
pref:0.7/0.3, constant
pref:0.7/0.3, burst
Average frequency
Density of insertions
Position
1e+06
2e+06
3e+06
4e+06
selection
s(HR)=-0.005, s(LR)=-0.0005
s(HR)=s(LR)=-0.0001
