# Supplementary material for: Disentangling the determinants of transposable elements dynamics in vertebrate genomes using empirical evidences and simulations
Source: PLoS Genet. 2020 Oct 5;16(10):e1009082. doi: 10.1371/journal.pgen.1009082 (PMC7561263; doi:10.1371/journal.pgen.1009082)
Supplement: S13 Fig — We simulate a 4Mb fragment, assuming the following unscaled parameters (see Methods for details about scaling): a stable effective population size of 1 million individuals, a mutation rate of 2.1.10−10/year, high recombination in the first and last Mb (r = 2.10−10 /year), low recombination in the 2 Mb in the middle (r = 2.10−11 /generation). Linked selection is modelled by introducing 10% of deleterious mutations with 2Nes = -10 in non-coding regions and 70% of deleterious mutations with 2Nes = -100 in coding regions. We assume that there are 10 TE progenitors in the whole genome that can jump P generations/genome (constant rate). We also model bursts of transposition where the probability of jumping is 100X higher, but transposition occurs during a lapse of 100,000 years, deviating from transposition-drift balance. We also add an insertion bias Q to model preferential insertion in regions of high recombination. (PDF) [file pgen.1009082.s013.pdf]

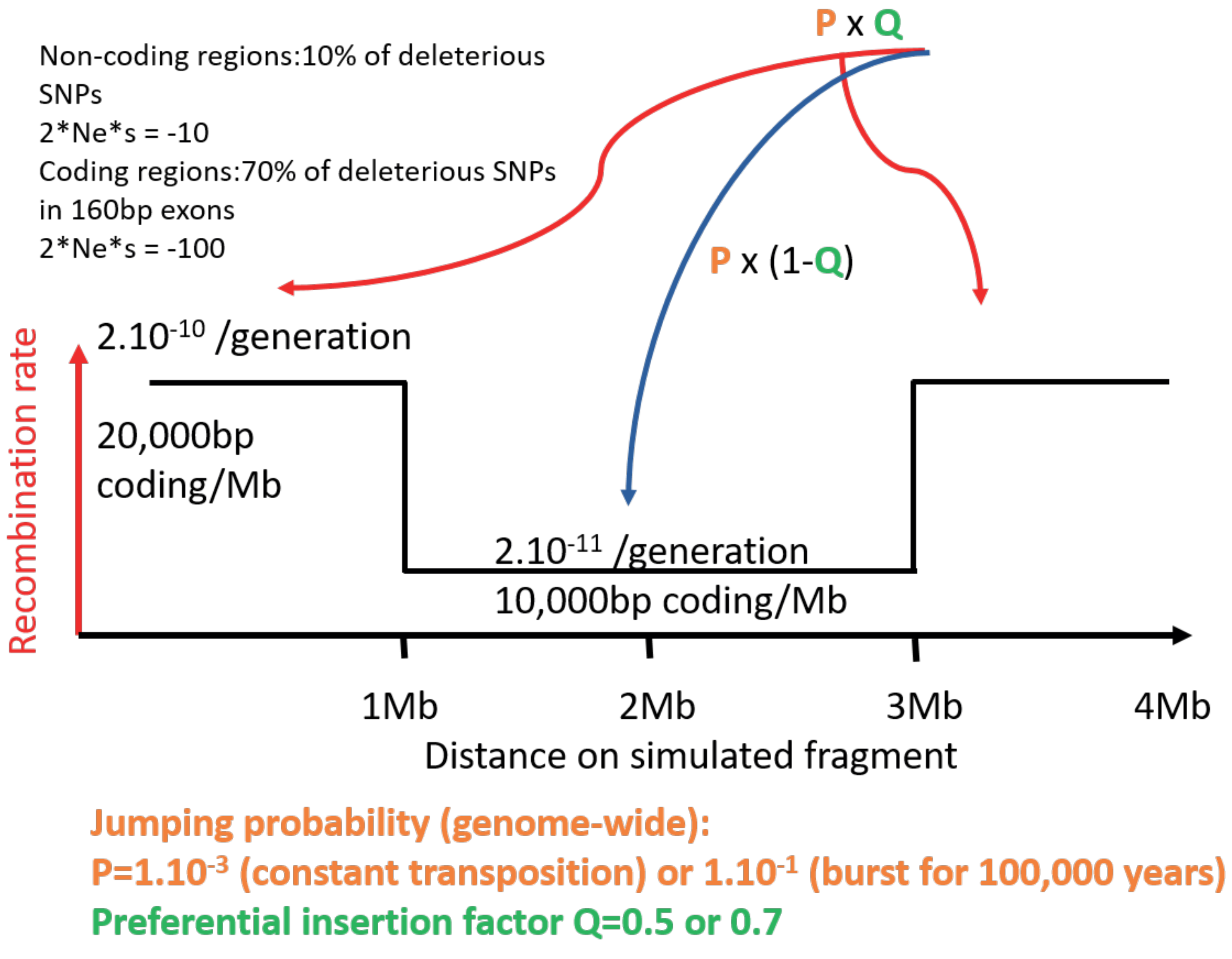

P x Q
Non-coding regions:10% of deleterious SNPs
2*Ne*s = -10
Coding regions:70% of deleterious SNPs in 160bp exons
2*Ne*s = -100
P x (1-Q)
Recombination rate
2.10^{-10} /generation
20,000bp coding/Mb
2.10^{-11} /generation
10,000bp coding/Mb
1Mb
2Mb
3Mb
4Mb
Distance on simulated fragment
Jumping probability (genome-wide):
P=1.10^{-3} (constant transposition) or 1.10^{-1} (burst for 100,000 years)
Preferential insertion factor Q=0.5 or 0.7
