# Supplementary material for: Disentangling the determinants of transposable elements dynamics in vertebrate genomes using empirical evidences and simulations
Source: PLoS Genet. 2020 Oct 5;16(10):e1009082. doi: 10.1371/journal.pgen.1009082 (PMC7561263; doi:10.1371/journal.pgen.1009082)
Supplement: Attachment — Submitted filename: Answers_Reviews_PloS_Genetics.docx [file pgen.1009082.s015.docx]

Yann Bourgeois

School of Biological Sciences, University of Portsmouth, Portsmouth PO1 2DY, UK

[yann.x.c.bourgeois@gmail.com](mailto:yann.x.c.bourgeois@gmail.com)

[yann.bourgeois@port.ac.uk](mailto:yann.bourgeois@port.ac.uk)

Portsmouth, 16th of August 2020

To the editorial board of *PloS Genetics*,

Dear Editors,

Please find enclosed our revisions for the manuscript entitled “**Disentangling the determinants of transposable elements dynamics in vertebrate genomes using empirical evidences and simulations”**. We answered to all reviewers’ comments. We added several elements to our SLiM models, such as variable density of functional elements and positive selection. We clarified the Introduction and Figures. We also provide more details about candidate insertions for positive selection.

We hope that you will find this revised manuscript of interest and suitable for publication in *PloS Genetics*.

Yours sincerely,

Yann Bourgeois, Ph.D., and co-authors.

########################################################################

Reviewer's Responses to Questions

**Comments to the Authors:
Please note here if the review is uploaded as an attachment.**

Reviewer #1: I think this is a very interesting work shedding light on the dynamics of TEs in non-mammals vertebrates by combining real data with simulations. The authors provide the readers with the scripts to perform the simulations so I think this can also be very useful for the community. I have a few suggestions and questions.

**Thank you. We do hope our work be useful for further investigations.**


Line 70. References should be added for the so called "alternative model"

**We added a reference to a review from 2018 by Irina Arkhipova in MBE (35(6):1332–1337), which examines the dynamics of TEs in the light of Kimura’s neutral theory.**

Line 179. The authors used LINEs as a synomym of nLTRs-RT in the text while in the figures they use mostly (not always) nLTRs-RT. For consistency, I would always used the same nomenclature or specify that they are used interchangeably.

**We clarified at several places in the text. We now use nLTR-RT in the text and figures. We refer to LINEs only when examining the relationship between length and recombination, since we exclude *Penelope* in that specific analysis.**

Figure 1. Consider plotting "private", "shared with green anole only" and "shared with outgroup" in that order.

**This is indeed more logical.** **We edited as suggested.**

Figure 2 will benefit from adding the class of TE nearby the letters, e.g. A)nLTR-RTs; B) SINEs etc...

**We edited as suggested.**

Line 214. The impact of recombination on TE frequencies has been studied before, see for example Petrov et al 2011, Kofler et al 2012, Kapun et al 2020.

**We acknowledge this, but we simply wanted to explain to the reader what the purpose of this specific analysis was. We already cite Petrov et al. 2011, Kofler et al 2012 in other parts of the article. We added the Kapun et al reference (L489).**

***“We obtained intriguing results for SINEs, DNA transposons such as Tc1/Mariner, and short nLTR-RTs. Under the ectopic recombination hypothesis [36,47], which is usually invoked to explain genome-wide patterns of TE diversity, TEs tend to be removed from regions of high recombination through purifying selection. Such correlations have been commonly observed for several TE families in fruit flies and other vertebrates [7,36,48,49].”***


Line 226. It is not clear to the reader whether the "differentiation metrics" in figure 3 are calculated with SNPs or with TEs. The figure legends only mentions TEs but this is not clear in the text. Line 232 starts with "Linked selection should have a similar effect on TEs" which leds the reader to think that the previous metrics where calculated for SNPs. If that were the case, figure legend and the figure itself should be edited (Density of polymorphic insertions should be on top of the TE part of the figure).

**We edited the figure and its legend to make it clear that differentiation and diversity metrics are computed for SNPs (left side of the figure), then compared to TE density (right side of the figure).**

Line 226. Moreover, is not clear whether results for the "differentiation metrics" refer to polymorphic, fixed, or both types of variants.

**First, we examine the correlations expected between several summary statistics based on SNP data to confirm a likely effect of purifying selection, then examine TE densities. We edited this part so the presentation is clearer (L236):**

***“Studies focusing on SNPs have revealed that regions of low recombination display lower diversity and stronger differentiation between populations due to the effects of linked selection [34,35]. First, we tested whether typical signals of linked selection could be observed along the genome by examining correlations between recombination rates, derived allele frequencies, and absolute (d_XY_) and relative (F_ST_) measures of differentiation computed over SNP data in non-overlapping 1Mb windows (Figure 3). We focused on the six main autosomes of the green anole. If linked selection shapes genomic diversity along the genome, there should be 1) positive correlations between diversity indices (average derived allele frequency, d_XY_) and recombination, 2) negative correlations between differentiation measures (F_ST_) and recombination, 3) consistency in genomic regions displaying high or low values for F_ST_ or d_XY_ across all pairwise comparisons. This is in line with our observations, with mostly positive correlations between recombination rate, diversity and absolute divergence for all pairwise comparisons between the five genetic clusters (Figure 3). Pairwise relative measures of differentiation (F_ST_) were negatively correlated with recombination rate, d_XY_, and derived allele frequencies, which is consistent with a role of linked selection reducing diversity in regions of low recombination across all genetic clusters. Indices of differentiation comparing CA or GA with other populations were less correlated with indices of differentiation estimated between pairs of clusters from Florida, suggesting a role for recent expansion in blurring the expected correlations.***

***Then, we examined densities of polymorphic and fixed TEs across four main categories of TEs (Figure 3). Assuming they are nearly neutral, linked selection should have a similar effect on TEs as on SNPs. The stronger Hill-Robertson interference observed in regions of low recombination should lead to a lower number of polymorphic there. On the other hand, it is generally assumed that rates of ectopic recombination increase with crossover rates. In that case, elements involved in ectopic recombination should be under strong purifying selection, slowing the accumulation of TEs in regions of high recombination compared to regions of low recombination. This should result in decreasing densities of both polymorphic and fixed elements as recombination increases. TE densities were positively correlated with recombination rate, diversity and relative measures of differentiation for SINEs and DNA transposons. Correlations were weaker for nLTR-RTs, and almost absent for LTR-RTs. The density of fixed LTR-RTs even followed an opposite pattern, with more fixed insertions in regions of low recombination and high F_ST_. For fixed nLTR-RTs, correlations were weak or absent. This suggests that purifying selection against LTR-RT and to some extent nLTR-RTs may explain the variation in their local abundance and diversity.”***

Line 253, text in this paragraph and Figure 4 nomenclature is misleading. For example, in Figure 1 DIRS are included in LTR-RT class while in Figure 4 the legend reads LTR-RT and DIRS as if they were two different categories.

**Thank you for spotting this. This is a remnant from a previous version where we considered applying another nomenclature (Wicker et al. 2007, Nat. Rev. Genetics). For the sake of simplicity, we include *DIRS* in LRT-RTs.**

Line 278. Mention in the text that the average frequency is for polymorphic insertions.
**Done (L310):
*“This should result in reduced frequency of polymorphic insertions and abundance of elements in regions of high recombination and high gene density.”***

Line 279. Mention here that correlations for density are done separately for polymorphic and fixed insertions.
**Done (L311):
*“To test whether TEs from different clades followed this predicted pattern, we assessed whether their average frequency, density of polymorphic insertions, and density of fixed insertions, varied with the recombination rate (Figures 5, 6, 7, Table 2).”***

Line 298. The relationship between TE size and recombination applies to all TE classes not only to LINEs (see e.g. Petrov et al 2011).
**We replaced “LINEs” with “TE insertions”.**

Line 325. I am curious to know why the authors chose the percentage (10%) of the SNPs to be under negative selection. Also why they simulated a 1+2+1 region with high+low+high recombination instead of e.g. 2+2. Maybe these small details can be added to the material and methods?
**We initially chose this proportion and selection coefficients because they mimicked the changes in SNP frequency and diversity we observed. There is little information about the actual proportion of functional elements in the green anole, and we considered that 10% of sites under purifying selection was reasonable (especially given the rather weak selective coefficient N*s = 10 that we applied). Since our goal was mostly to obtain qualitative results about the trends to be expected, we kept these parameters for our simulations. We chose this 1+2+1 configuration mostly for the sake of illustration: U-shapes are easy to visualize in Figure 9.

We took the opportunity of this revision to enrich our model, and now incorporate variable density of functional elements (short exons accounting for 10,000bp in regions of low recombination and 20,000bp in regions of high recombination). TEs that fall in an exon are strongly deleterious, with a selective coefficient of -1 (fitness of 0). To obtain a perhaps more accurate distribution of fitness effects in these functional region, we used recent results about dN/dS in several anole species, which suggest that about 15% of non-synonymous mutations may be near-neutral (see box 2 in Eyre-Walker & Keightley 2007, Nature Reviews Genetics).We considered that 25% of coding sites were synonymous.**

**To better illustrate how the choice of selective coefficients may change our results, we now include in the Supplementary Material simulations using various selective coefficients on SNPs, using 1 or 10% of mutations under purifying selection in non-coding regions, and incorporating positive selection in coding sequences. The results are consistent with the expectations: reducing the strength of purifying/positive selection on SNPs erodes the differences in frequency and density for “short” TEs. We note that including even a rather low proportion of sites under positive selection leads to strong interference effects.**

**We added these precisions in the Methods and Results section, and updated our scripts (**[**https://github.com/YannBourgeois/SLIM_simulations_TEs/blob/master/modelling_TEs_with_exons_and_positive_selection.txt**](https://github.com/YannBourgeois/SLIM_simulations_TEs/blob/master/modelling_TEs_with_exons_and_positive_selection.txt)**).**

Figure 9. Legend does not explain what A and B are in the figure. The discontinuos read and blue lines that appear in the first set of plots are not explained either.
**We added precisions to the legend, which now reads:**

***“Figure 9: Summary of simulations of TEs using SLiM3, using parameters realistic for the NEF cluster. Eight diploid individuals were sampled to mimic our sampling scheme. Boxplots correspond to the results obtained over 100 simulations of a 4Mb fragment, divided into three regions of 1, 2 and 1 Mb. The first and last Mb correspond to regions of high recombination and high density of functional coding sites (respectively 10 times and 2 times higher than in the 2Mb central region). Coefficients of selection and other parameters are scaled using an effective population size of 1000 instead of 1,000,000 to reduce computation time (see Methods). 2N_e_s = -10 for 10% of non-coding sites and 2N_e_s = -100 for 70% of coding sites. Blue and red dotted lines correspond to average derived SNP frequencies in regions of low and high recombination respectively. A: model with constant transposition and no preferential insertion; B: model with a burst of transposition. C and D are the same than A and B respectively, but include preferential insertion in regions of higher recombination such that 70% of new elements go into these regions. TEs that fall in coding regions are strongly deleterious (selection coefficient 2Ns=-2000).”***

Line 391. Reference to Gautier 2015 where XtX and eBPis is described.

**We added the reference.**

Line 398 I would suggest to mention here the three approaches used to identify positive selection and reference their publications.

**We now mention these approaches and added the references.**

Table 2. I would suggest to not use the abbreviations for the dominant process (process is missing an "s" in the table) because there is enough space to add the process itself, which facilitates the reading. Some abbreviations might be used e.g. "purifying selection against ectopic recombination" could be "purifying sel ectopic rcb" or something similar if needed (It looks like there is enough space to add the whole information).
**We agree, and modified the table as suggested.**

Line 430 Can the positive correlation between recombination rate and average frequency and density of polymorphic elements be explained by an enrichment of short lenght elements in the SINEs, and DNA transposons such as Tc1/Mariner groups of TEs? The correlation between recombination rate and average frequency is expected for long TE copies (see for example Petrov et al 2011).

**We fully agree with this, and edited the sentence as follows to make it more explicit (L503):
*“We note that these elements tend to be quite short in length, which may make them nearly neutral, and therefore more likely to be impacted by linked selection.”***

Line 555 Although I do not disagree with the authors in their approach, I do think it might be way too stringent. How many TEs appear to be under positive selection with each individual test or according to two of the tests? I am curious to know these numbers, maybe they can be given and discussed.

**We agree, and have relaxed our criteria. We now provide the whole list of elements, their frequencies, and their overlap with other tests. Note that we also relaxed a constraint on the minimal 50% overlap between outlier windows from LSD/BAYPASS and diploS/HIC, which rises the number of TEs overlapping between all three tests to 6. We edited the Supp. Methods accordingly and edited Tables, Methods, Results and Discussion to reflect this.**

Reviewer #2: In this paper, Bourgeois et al. investigated the dynamics of segregating and fixed transposable element copies in the genome of Anolis carolinensis. Different types of elements show contrasting patterns of correlations with recombination rate. This relationship may also vary between the fixed and the polymorphic elements within a given group. These results may be explained by demography, linked selection and/or preferential insertions. The results also show a lack of selection against short insertions and the authors used very careful approaches to identify limited signatures of adaptive TE insertions in recent evolutionary history.

The manuscript is well-written and certainly of interest to the readership of PLoS Genetics. However, some points require minor revision for clarity – especially related to the high density of functional sites in high-recombination regions and the regulatory potential of short vs. long TE insertions. My detailed comments are as follows (ordered by line number):

**Thank you for your interest and useful comments. We hope you find our answers satisfying.**

1. General comment on the usage of linked selection: Throughout the manuscript it is unclear if linked selection includes or excludes the effect of selection on the TEs themselves.
 **We consider linked selection as the effect of any deleterious/positively selected variant on regions nearby.** **We make the distinction between linked selection and direct selection on TEs due to ectopic recombination/falling in functional regions (e.g. exons).
We clarify this issue in the Introduction (L76)**

***“In addition, stochastic processes may not be constant along the genome. For example, recent investigations have highlighted the importance of recombination rates in shaping genomic diversity, due to the effects of selection at linked sites. Because of Hill-Robertson interference, regions near a selected site see their genetic diversity drop, an effect that increases in regions of low recombination [14]. This drop may not only affect nucleotide diversity, but also TEs and other structural variants.”***

2. General comment on simulation: In SLiM 3 a recombination map is set up at the beginning of a run and each generation a single recombination map applies for all individuals (see page 566 of the SLiM manual). This could potentially be an issue here and something the authors should carefully consider.

**Here, we simulate a single panmictic population, and we consider that individual variation in recombination rates along the simulated region is unlikely to be a major issue. Modifying the recombination map at the individual level during the simulation through recombination() callbacks may be useful for studies examining how TEs accumulate in regions that stop recombining (for example sex chromosomes degeneration or inversions). This is however beyond the scope of the present paper.**

3. Line 31: Please replace “TEs frequencies” with “frequency of TEs”.
**Done**

4. Line 49: Please replace “he” with “the”.
**Done**

5. Line 50: Please change “diversity” to “diversities”. **Done**

6. Line 68: How can expansion be independent of transposition? Through segmental duplications? A clarification of a stochastic expansion here would be good and separating genetic drift affecting TE copies and host.

**This sentence was clumsy; we meant demographic changes such as expansion and bottlenecks (L67).

*“An alternative model would posit that TE diversity be driven by stochastic events of population size changes in the host and drift that are independent of intrinsic TE properties such as selection or transposition (neutral theory) [6].”***

7. Line 79: Is it correct to say “Hill-Robertson interference and hitchhiking”, considering that hitchhiking is a form of HRI?

**We agree, and keep “Hill-Robertson interference” since it is the more general term.**

8. Lines 101-102: Could you discuss here the presence of promoters and enhancers depending on TE structure and mechanism?
 **We now mention that TEs vary in terms of regulatory content (L115): *“Since TEs vary in their mode of transposition, length, regulatory content and structure, it is likely that the effect they have on host fitness and how they are in turn affected by host-specific response will differ.”***

9. Line 150 and other appropriate sections: In the beginning of the Results and Figure 1, it would help the reader if the bottlenecked populations are explicitly mentioned or highlighted. Maybe a bracket around GA and CA as "Northern clades" would help? Please also mention which population the anole reference genome belongs to, in case the reader wonders about potential reference biases.
 **We edited Table 1, Figure 1 and the text to reflect this (L169)**

***“Two of these genetic clusters (GA and CA, referenced as Northern populations) went through a bottleneck 100,000 years ago. Note that the individual used to build the reference genome was sampled in South Carolina, which places it in the Northern populations [31].”***

10. Line 154 and other appropriate sections: Conceptual question regarding cut-and-paste DNA transposons - how are allele frequencies dealt with for these?
 **We cannot determine whether DNA transposons at different sites are actually the same element that cut and pasted in different individuals using MELT. We mostly use information about presence/absence at a given site, treating DNA transposons the same way as other elements. This is enough for our purpose, since we are not directly interested in the origin of a particular insertion, but rather in the broader diversity over large genomic regions. See also our answer to point 21.**

11. Line 167: Please provide a reference for "low homoplasy of TE insertions".
**We added references.**

12. Line 177: Please replace “population” with “populations”.
**Done**

13. Line 179: Please insert "heterozygous" before "TEs".
**We actually replace by “polymorphic”, since we are considering the process at the population scale.**

14. Line 186-190: I admit it is not clear to me whether the high numbers of heterozygous TEs in outgroups are due to technical reasons (more non-reference insertions with more phylogenetic distance) or biological (e.g., reflecting differences in TE expansion or host population sizes)? I suggest double-checking for a potential role of coverage difference in the number of identified heterozygous TEs (i.e., more false negatives with lower coverage). If plotting the coverage per individual vs. the number of identified heterozygous TEs, is there a positive correlation?
**We acknowledge that it remains difficult with short Illumina reads to discard phylogenetic distance completely. Nevertheless, the depth of coverage for the two outgroups is not particularly low compared to the average of samples (~12.2X and 13.0X for *A. allisoni* and *A. allisoni* respectively, average for all samples=11.6X). The high number of heterozygous TEs in the outgroups is not a systematic effect (for example in the case of *Gypsy* elements we observe an opposite pattern).**

15. Line 205: Can you comment on the size of LTRs (larger than LINEs) and the expectation that each LTR insertion (full-length and solo) should contain regulatory elements, while 5'-truncated LINEs should not contain these?
**We added the following sentences in the Discussion (L596)

*“Full-length LTR-RTs are very long elements (~5,000bp) in the green anole, that may be strongly deleterious under the ectopic recombination model. In addition, they harbor regulatory motifs that may increase their deleterious effects near coding regions”***

16. Line 235: What is meant with the phrase "purifying selection against elements through ectopic recombination"? Do you mean that TEs are selected against in regions where they would otherwise lead to ectopic recombination?

**We clarify as follows (L257):

*“The stronger Hill-Robertson interference observed in regions of low recombination should lead to a lower number of polymorphic TEs there. On the other hand, it is generally assumed that rates of ectopic recombination increase with crossover rates. In that case, elements involved in ectopic recombination should be under strong purifying selection, slowing the accumulation of TEs in regions of high recombination compared to regions of low recombination.”***

17. Line 235: Has it been proven that the rate of ectopic recombination is positively correlated with the population-effective recombination rate or crossover rate? Barrón et al. (2014; Population Genomics of Transposable Elements in Drosophila. Annual Review of Genetics, 48(1), 561–581. https://doi.org/10.1146/annurev-genet-120213-092359) states that it’s assumed. If you know of any source that have shown such a relationship, it would be helpful to the field to provide. Otherwise it would be good to clarify that it’s an assumption.

**It seems mostly to be an assumption indeed. We clarified accordingly: *L108: For example, it is often assumed that higher recombination rates may result in higher rates of ectopic recombination, making repetitive elements more deleterious in regions of high recombination (e.g. [7,15]).

L259: On the other hand, it is generally assumed that rates of ectopic recombination increase with crossover rates. In that case, elements involved in ectopic recombination should be under strong purifying selection, slowing the accumulation of TEs in regions of high recombination compared to regions of low recombination.***

18. Line 244: Please insert "the variation in" before "their".
**Done**

19. Lines 245-249 and other appropriate sections: Unclear what is meant in this paragraph - if there is a positive correlation of exon density and recombination rate, doesn't this higher density of functional elements explain the low density of LTRs and LINEs in these regions? Please revise this paragraph for clarity and consider adding this point in other appropriate sections.

**We agree that both higher recombination rates and higher density of functional elements may explain the lower density of long elements in regions of high recombination. Nevertheless, exons account for 1 to 2% of our 1Mb genomic windows, and the fact that short LINEs display opposite patterns compared to long ones suggest that ectopic recombination may play a major role. We edited the text as follows (L332):
 *“This may be due to the fact that the odds of homologous recombination rise with the length of homologous fragments [38], or because longer elements contain promoter sequences that may have more deleterious effects on nearby genes.”***

20. Lines 250-252: Unclear what is meant with this paragraph - is the point here the similarity in densities between TE groups, or between host populations? Please revise this paragraph for clarity. One of the confusing aspects is the use of terms like "genetic groups" and "clades" in slightly different contexts across paragraphs.

**We mean host populations. We rephrased, and try to make the distinction between host populations and TE clades clearer across the text (the term “clade” should now only refer to TEs, and “populations” or “clusters” to demography).**


21. Lines 272-275: What about the possibility that occasional cut-and-paste would reduce the frequency of (nearly) fixed DNA transposon insertions?

**We now acknowledge this possibility in the text (L303). However, this seems unlikely to result in detectable changes in frequency for insertions that are nearly fixed, given that we examine 8 diploid individuals sampled from a population with large effective size (N_e_~1 million diploid individuals).**

***“Because DNA transposons replicate through a cut-and-paste mechanism, it may happen that some insertions be removed from a given insertion site. Nevertheless, the large effective population sizes considered here would make any substantial impact of occasional cut-and-paste extremely limited in terms of allele frequency.”***

22. Line 276: See above comment #17.

**We clarify (L307):**

***“TEs involved in ectopic recombination should be subject to purifying selection, becoming stronger in regions of high recombination. In addition, the higher exon density in these regions (Figure S6) may increase the odds that these TEs alter gene expression. This should result in reduced frequency of polymorphic insertions and abundance of elements in regions of high recombination and high gene density.”***

23. Line 285: I agree that ectopic recombination would be a strongly deleterious effect of TEs in high-recombination regions, however, considering that exon density also correlates with recombination rate, what about the possibility that large TEs containing regulatory elements are particularly deleterious in regions with many genes?
 **We agree with the reviewer, and make this more explicit (L307) See also our answer to comment 28: we include exon density in our models.**

24. Line 299: See comment #15, what if longer LINEs have regulatory elements in their 5' ends that shorter, truncated LINEs don't have?

**See answer to Comment #15. We also edited as follows (L332)**

***“This may be due to the fact that the odds of homologous recombination rise with the length of homologous fragments [38], or because longer elements contain promoter sequences that may have more deleterious effects on nearby genes.”***

25. Line 301: The assumption for this conclusion is that average TE copy age is uncorrelated to average TE copy length, however, this is not tested here and might not be true. The rate of deletion may also be correlated with recombination, see Nam and Ellegren (2012; Recombination Drives Vertebrate Genome Contraction. PLoS Genetics, 8(5), e1002680. https://doi.org/10.1371/journal.pgen.1002680). As a control, did you test whether any elements are 3'-truncated (instead of being 5’-truncated as expected from the insertion mechanism)?

**Previous studies on LINEs in the green anole did not reveal substantial truncation in 3’, but we acknowledge that they were based on a rather small subset of LINEs (Tollis and Boissinot 2013). We provide a control by examining for the different TE clades the distribution of starts and ends on their respective consensus (given in the MEINFO field in the VCF output by MELT). As illustrated by the new Sup. Figure 7, there is substantial truncation at the 5’ end, but almost none at the 3’ end for TEs. We add the following sentence to clarify (L 357):**

***“These results could also be explained by a higher rate of deletion in TE insertions located in regions of high recombination [39], or older elements containing more deletions. However, an examination of the start and end coordinates of insertions on their consensus did not reveal any substantial truncation at the 3’ end (Figure S7), which would be expected if deletion occurred once the element is already inserted. We only observed truncation at the 5’ end, which is consistent with truncation during the insertion process.”***

26. Line 313: What does ">30% max length" mean?
**We clarify (L349):**

***“For long LINEs (>30% of the maximum length of their specific clade), we observed strong negative correlations between TE frequency, the density of fixed insertions, and recombination.”***

27. Line 316: I advise the authors to interpret this more cautiously as recombination may create shorter elements through random deletion (Nam and Ellegren, 2012). This could be especially true for L2s which contain a recombination hotspot motif, at least in humans (McVean, 2010; What drives recombination hotspots to repeat DNA in humans? Philosophical Transactions of the Royal Society B: Biological Sciences, 365(1544), 1213–1218. <https://doi.org/10.1098/rstb.2009.0299>).

**Thank you for these references. Please see answer to 25 (L 357).**

***“These results could also be explained by a higher rate of deletion in TE insertions located in regions of high recombination [39], or older elements containing more deletions. However, an examination of the start and end coordinates of insertions on their consensus did not reveal any substantial truncation at the 3’ end (Figure S7), which would be expected if deletion occurred once the element is already inserted. We only observed truncation at the 5’ end, which is consistent with truncation during the insertion process.”***

28. Lines 321-322 and other appropriate sections: Would it be possible to take into account the density of functional elements (exon density, for example)? After all, the strength of purifying and linked selection don't only depend on the recombination rate, but also on the distance of a TE insertion to a functional site under purifying selection.

**We now incorporate this aspect in the simulations, which also has the advantage of providing a (perhaps) more realistic distribution of fitness effects (most of these distributions having been obtained from coding regions in vertebrates).**

**We took the opportunity of this revision to enrich our model, and now incorporate variable density of functional elements (short exons accounting for 10,000bp in regions of low recombination and 20,000bp in regions of high recombination). To obtain a perhaps more accurate distribution of fitness effects in these functional region, we used recent results about dN/dS in several anole species, which suggest that about 15% of non-synonymous mutations may be near-neutral (see box 2 in** Eyre-Walker & Keightley 2007, Nature Reviews Genetics).**We considered that 25% of coding sites were synonymous.**

**To better illustrate how the choice of selective coefficients may change our results, we now include in the Supplementary Material simulations using various selective coefficients on SNPs, using 1 or 10% of mutations under purifying selection in non-coding regions, and incorporating positive selection in coding sequences. The results are consistent with the expectations: reducing the strength of purifying/positive selection on SNPs erodes the differences in frequency and density for “short” TEs. We note that including even a rather low proportion of sites under positive selection leads to strong interference effects.**

**L729:**

***“Because regions of higher recombination tend to display more exons (Figure S6), we*** ***assigned to regions of low and high recombination 10,000 bp and 20,000 bp of coding sequences per Mb respectively. We simulated 160 bp exons (close to the average length of exons in the green anole) that were randomly placed until the desired density was reached. To explore the effects of linked selection due to deleterious and positively selected sites, we varied the proportion of nucleotide mutations under selection. In exons, we kept the proportion of new deleterious point mutation at 70% in all simulations, which seems reasonable given that dN/dS in anoles are about 0.15 [77], suggesting that most substitutions at non-silent sites are deleterious (see box 2 in [78]). To obtain the results shown in Figure 9, we assumed that deleterious mutations in exons would display a strong effects on fitness, with 2N_e_s = -100 (s = 5.10^-5^). Of all new point mutations in non-coding regions, 10% were deleterious with 2N_e_s = -10 (s = 5.10^-6^). There is not much information about the fitness effects of new mutations in non-coding sequences in vertebrates in general, and the green anole in particular. However, our estimate seems conservative given that in mice and humans, about 20-40 % of mutations in conserved regions may have an s > 3.10^-4^ [79]. To explore further how varying selective coefficients may impact our results, we examined results from simulations with 2N_e_s = -40 or 2N_e_s =-1 in 10% of non-coding sites, and 2N_e_s = -400 or 2N_e_s = -10 in coding regions (Figure S8.and S9). We also examined a case with almost no purifying selection on nucleotides, with only 1% of non-coding sites being under purifying selection (Figure S10). We acknowledge that positive selection may also play a major role in reducing diversity, and also explored how adding positive selection to the latter model with little purifying selection on nucleotides may restore the correlations we observed with strong purifying selection. We added positively selected substitutions, with 1% and 5% of new substitutions in coding regions with 2N_e_s = +10 (0.1% and 0.5% in non-coding regions, Figures S11 and S12 respectively).”***

29. Line 324: Here you write that you “built a simple model” though the core structure of your model greatly resembles (i.e. equal variable names with identical capitalizations) recipe 14.12 in SLiM3. I agree that you have modified the original recipe and created your own specific model which could motivate this kind of wording, but please also acknowledge the recipe creator.

**We agree. This omission was not acceptable. We added the following sentence (L 721):

“*We modified a preexisting recipe (14.12) provided by Benjamin Haller and Philipp Messer”***

30. Line 386: In line with an earlier comment, please consider whether the TE insertion of interest can actually contain a regulatory element or not (e.g., severely 5'-truncated LINEs).
 **Thank you for this suggestion. We provide a new table that provides more information about the selection tests and gives the length of elements.**

31. Lines 452-454: I fear I don't follow here - wouldn't high recombination reduce the effect of Hill-Robertson interference? Could the reduced number of fixed TEs result from the high density of functional sites (exon density)?

**We do mean that higher recombination limits Hill-Robertson interference and may accelerate the *fixation* of *near-neutral* TEs in regions of low recombination, while decreasing the density of *polymorphic* insertions in regions of low recombination. Hill-Robertson interference can be approximated by a local reduction in the effective population size (“N”), which reduces the time during which an allele remains polymorphic. The probability of fixation of a new near-neutral mutation (proportional to 1/N, with N the “local” effective population size) is higher in regions of low recombination. On the other hand, the time to fixation is longer (proportional to N) in regions of high recombination. This leads to more polymorphic insertions in regions of high recombination, but less frequent fixation. In other words, higher recombination leads to more insertions that lie in the middle of the frequency spectrum. Direct selection against TEs in regions of high recombination/high density of functional elements would reduce the density of both polymorphic and fixed sites in these regions (since they rarely reach appreciable frequencies and go extinct most of the time, as long as Hill-Robertson interference is not too strong).**

**Note that in our new set of simulations, we add a scenario with strong Hill-Roberson interference (N*s=-400 in exons, and N*s=-40 in 10% of non-coding sites). We observe that the number of polymorphic insertions decreases in regions of low recombination, to the extent that for “full length” TEs, there is no substantial difference in the density of polymorphic insertions between regions of high and low recombination (see left upper panel in “Density of polymorphic insertions”).**

32. Line 459: Note that the study cited was not able to differentiate between full-length LTRs and solo-LTRs, however, it is possible that frequent solo-LTR formation under high recombination rates will allow these to accumulate in high-recombination regions. If space allows, please consider mentioning this point here.

**Done: L 519:
*In the case of LTR-RTs, it may also be that higher recombination rates increase the frequency of solo-LTR formation, potentially limiting their deleterious effects.***

33. Lines 463-464: The point of LTR accumulation being ancient in birds is incorrect, as recently shown by two population-level studies (ref. 44 and Weissensteiner et al. 2020 The population genomics of structural variation in a songbird genus. biorxiv doi:10.1101/830356). Instead, most of the recent TE activity in songbirds appears to be LTR families. Please revise accordingly. What if all these LTRs are solo-LTRs in birds but less often so in anole?
**We include this possibility in our discussion. We actually meant that LTR accumulation may be more recent in the green anole than in birds (L525):

*“We note that these families may be older in birds than in the green anole, having accumulated between 55 and 33 Mya [54], while a substantial proportion of these elements display less than 1% divergence from their consensus in green anoles (see repeat landscape at*** [***http://www.repeatmasker.org/species/anoCar.html***](http://www.repeatmasker.org/species/anoCar.html)***, last accessed 25/03/2020). It is therefore possible that purifying selection has had more time to remove the most deleterious insertions in birds, increasing the signal of preferential insertion that may be masked in the green anole. It may also be that LTR-RT elements produce more frequently solo-LTR in birds than in lizards, which would make them less deleterious and more subject to drift and linked selection.”***

34. Line 489: A reference to this statement would be helpful for the reader or some numbers from the present data.
**We edited as follows (L553):

*“We note however that L1s are substantially longer than other LINEs (Figure 8; average length of 3700 bp, 1194 bp and 1448 bp for L1, L2 and CR1 respectively for all insertions in the dataset, Wilcoxon tests, all P<2.2.10-16), which limits our power to study short elements.”***

35. Line 570: Please replace "details" with "detailed".
***Done***

36. Line 592 and other appropriate sections: Two key points of the "Calling TEs" methods section should be mentioned in the Results/Discussion sections (because most studies on this topic should do this but don't): 1) that both reference and non-reference insertions were analyzed and 2) only insertions with no missing data for genotypes were analyzed.
 **Done (L167)
 *A total of 339,149 polymorphic TE insertions with no missing genotype were recovered from resequencing data obtained from 28 anoles, including the five genetic clusters identified in previous studies [26,27]. This included both reference and non-reference insertion.***

37. Line 628: Unclear what densities and frequencies refer to here - relative to genomic distribution or to the sampled populations?

**Densities are relative to genomic distribution, while frequencies refer to the average allele frequencies in a given population. L 692:**

***“We focused on three commonly used statistics to describe TE diversity in each genetic cluster: the density of polymorphic TEs along the genome, the density of fixed TEs along the genome, and the average frequency of polymorphic TEs in the host’s population.”***

38. Line 655: Please briefly describe in the text here what parameters are realistic for anoles.
 **Done (L722):
 *We simulated a 4Mb genomic fragment with parameters such as effective population size, exon density, mutation and recombination rates that were realistic for green anoles (Figure S7).***

39. Line 664. I get 1.10-3.5 to be ~0.72. Is this really the selection coefficient intended?

**We mean 10^(-3.5). In the original paper, selective coefficients are expressed in log10 units, and 20-40% of mutations have a log10(*s*)<-3.5. We now write 3.10^-4^, which is a clearer notation.**

40. Line 668: Is the assumption of 10 TE progenitors realistic given the many active families/subfamilies of TEs in anole?

**We precise that we mean 10 TE progenitors produced densities consistent with our observations *per TE clade*.**

41. Line 683: With the transposition-burst model, is mutation-selection-drift balance really achieved? Since mutations are only entering for a limited number of generations, this might
seems unlikely.
 **We completely agree with this comment, and actually wish to emphasize it: this scenario is a non-equilibrium case for TEs. L 759:
 *We also modelled bursts of transposition where P was set 100 times higher, but with transposition occurring only during a lapse of 100,000 years, starting 1,000,000 years ago. Note that in that latter case, TE insertions do not reach transposition-selection-drift equilibrium.***
 **Note however that we specify that we let the simulations run for 10,000 generations to reach mutation-selection-drift balance for nucleotide polymorphisms (L 771)**

42. Figure 2: Please consider showing AFS side-by-side barplots for easier readability.
 **Done.**

Reviewer #3: This is an interesting study addressing the landscape of polymorphism and fixation of TEs, with accompanying simulations, to make inferences about the factors influencing TE polymorphism in natural populations of Anolis lizards.

I found the study interesting, and the conclusions generally well supported. However I do have some concerns and suggestions that I think are well worth considering in a revised version:


1) the section on the 'visual inspection of allele frequency spectra' could do with some statistical tests (e.g. Mann-Whitney tests). I realize there's probably sufficient data for these to be generally significant, but worth demonstrating
 **We edited as follows (L 220):
 *This was reflected by systematically higher average allele frequencies in GA than in NEF (Wilcoxon tests, P <5.7.10^-12^ except for SINE3; P=0.03), the only exception being ACASINE for which no significant difference was observed.***

2) I think the introduction and various parts of the manuscript could do with a broader discussion of the effects of recombination on TEs. The intro focuses primarily on linked selection effects, but there are important alternatives that come up elsewhere in the manuscript, such as ectopic recombination and insertion preferences. Because recombination effects come up across the paper, I think a broader introduction to these issues is warranted. It would also be helpful to introduce alternative scenarios in the introduction, dependent on whether TE insertions are nearly neutral or subject to strong purifying selection.
 **This is in line with comments by reviewer 2, and we discuss more these mechanisms from the Introduction (L 83):**

***“In this work, we investigate three main factors that may impact TE distribution and diversity in the genome: direct selection on TEs, Hill-Robertson interference, and differences in their properties (e.g. preferential insertion). Many of these mechanisms make predictions about the correlation between recombination rate and diversity. For example, it is often assumed that higher recombination rates may result in higher rates of ectopic recombination, making repetitive elements more deleterious in regions of high recombination (e.g. [7,15]). This should result in negative correlations between TEs abundance/frequency and recombination. Hill-Robertson interference leads to shorter coalescence times in regions of low recombination. This may result in a faster fixation of neutral and slightly deleterious mutations, but also in lower polymorphism than in regions of high recombination [16,17]. At last, because recombination rate is often correlated with other genomic features such as exon density, DNA repair machinery, or open chromatin, variation in TE insertion mechanisms may be reflected in correlations between their density and recombination.”***

3) Page 9, line 232- related to point 2 above, some of the predictions being made should be contextualized more. e.g. saying that 'linked selection should have similar effect on TEs as on SNPs' is accurate if we are talking about effectively neutral TEs and effectively neutral SNPs. Since this ends up being a part of the interpretation, I think it's important to make this clear in the setup to the test. You bring up the 'on the other hand' immediately after, but I think for clarity it's worth emphasizing here that this is if TEs are effectively neutral.

**We edited the sentence to emphasize the near-neutrality of TEs in this context (L 256):**

***“Assuming they are nearly neutral, linked selection should have a similar effect on TEs as on SNPs.”***

4) page 8, line 245- Unless I'm very much misunderstanding, I think the first sentence should read: 'The higher abundance of some TE categories in regions of HIGH recombination', not low? Otherwise I'm very confused about this, since regions of low recombination DO seem to show weaker deleterious effects due to lower exon density

**This sentence is indeed confusing. LTR-RTs, long LINEs are clearly more abundant in regions of low recombination, which may be due to ectopic recombination and higher density of functional sites. Others are generally more abundant in regions of high recombination, but this is not due to lower density of functional elements in these regions. We reformulate as follows (L272):

*“The lower abundance of some TE categories in regions of low recombination was not explained by a higher density of functional elements that could increase their deleterious effects (Figure S6).”***


5) page 10, line 276- related to points above, all of a sudden the ectopic exchange model is introduced, now ignoring the potential countervailing effects of linked selection, which now come up as an alternative explanation for SINEs and DNA elements, even though linked selection effects were brought up already earlier as the 'primary expectation for TE polymorphic densities. Again, I think it's helpful to spell out more clearly distinct expectations for when TEs are under direct purifying selection (either due to ectopic recombination or a higher density of functional elements) and when they are effectively neutral, and how this could drive alternative results. Presenting these as two (three?) alternatives throughout the results and intro will be extremely helpful.
 **We agree, and recapitulate in the Introduction the different alternatives.**

**We also specify, L275:**

***“Then, we examined densities of polymorphic and fixed TEs across four main categories of TEs (Figure 3). Assuming they are nearly neutral, linked selection should have a similar effect on TEs as on SNPs. The stronger Hill-Robertson interference observed in regions of low recombination should lead to a lower number of polymorphic TEs there. On the other hand, it is generally assumed that rates of ectopic recombination increase with crossover rates. In that case, elements involved in ectopic recombination should be under strong purifying selection, slowing the accumulation of TEs in regions of high recombination compared to regions of low recombination.”***

TEs involved in ectopic recombination should be subject to purifying selection, becoming stronger in regions of high recombination. In addition, the higher exon density in these regions (Figure S6) may increase the odds that these TEs alter gene expression. This should result in reduced frequency of polymorphic insertions and abundance of elements in regions of high recombination and high gene density.

6) I really liked the simulation results and their connection to the data. One concern I had though, is that Ns=10 is still pretty weak purifying selection, especially for thoughts about the average effect of ectopic recombination. Can the authors add one more case, of Ns=100?

**We created new models and explored a bit more the effects of Hill-Robertson interference due to purifying selection (with examples of Ns reaching -400 in exons and -40 in non-coding regions), but also positive selection, and explored how the density of functional sites may change the results.**

7) I don't feel strongly, but from my sense 'lineage sorting' is usually not discussed in the context of linked selection, when we are not discussing two taxa and the effects of 'incomplete' or 'complete' lineage sorting. How about just referring to the effects of linked selection as 'shortening coalescent times'?
 **This is indeed more accurate, given that we are examining a single population. We edited as follows (L257):

*“The stronger Hill-Robertson interference observed in regions of low recombination should lead to a lower number of polymorphic TEs there.”***
